# SBDS-Deficient Cells Have an Altered Homeostatic Equilibrium due to Translational Inefficiency Which Explains their Reduced Fitness and Provides a Logical Framework for Intervention

**DOI:** 10.1371/journal.pgen.1006552

**Published:** 2017-01-05

**Authors:** Piera Calamita, Annarita Miluzio, Arianna Russo, Elisa Pesce, Sara Ricciardi, Farhat Khanim, Cristina Cheroni, Roberta Alfieri, Marilena Mancino, Chiara Gorrini, Grazisa Rossetti, Ivana Peluso, Massimiliano Pagani, Diego L. Medina, Johanna Rommens, Stefano Biffo

**Affiliations:** 1 INGM, National Institute of Molecular Genetics, “Romeo ed Enrica Invernizzi”, Milan, Italy; 2 DiSIT, University of Eastern Piedmont, Alessandria, Italy; 3 School of Biosciences, University of Birmingham Edgbaston Birmingham, United Kingdom; 4 Campbell Family Institute for Breast Cancer Research, University Health Network, Toronto, Ontario, Canada; 5 Princess Margaret Cancer Centre, University Health Network, Toronto, Ontario, Canada; 6 Telethon Institute of Genetics and Medicine (TIGEM)-Fondazione Telethon, Pozzuoli, Italy; 7 Department of Medical Biotechnology and Translational Medicine, University of Milan, Milan, Italy; 8 SickKids Children Hospital, Toronto, Ontario, Canada; 9 DBS, Università degli Studi di Milano, Milan, Italy; The University of North Carolina at Chapel Hill, UNITED STATES

## Abstract

Ribosomopathies are a family of inherited disorders caused by mutations in genes necessary for ribosomal function. Shwachman-Diamond Bodian Syndrome (SDS) is an autosomal recessive disease caused, in most patients, by mutations of the *SBDS* gene. SBDS is a protein required for the maturation of 60S ribosomes. SDS patients present exocrine pancreatic insufficiency, neutropenia, chronic infections, and skeletal abnormalities. Later in life, patients are prone to myelodisplastic syndrome and acute myeloid leukemia (AML). It is unknown why patients develop AML and which cellular alterations are directly due to the loss of the SBDS protein. Here we derived mouse embryonic fibroblast lines from an *Sbds*^*R126T/R126T*^ mouse model. After their immortalization, we reconstituted them by adding wild type *Sbds*. We then performed a comprehensive analysis of cellular functions including colony formation, translational and transcriptional RNA-seq, stress and drug sensitivity. We show that: 1. Mutant *Sbds* causes a reduction in cellular clonogenic capability and oncogene-induced transformation. 2. Mutant *Sbds* causes a marked increase in immature 60S subunits, limited impact on mRNA specific initiation of translation, but reduced global protein synthesis capability. 3. Chronic loss of SBDS activity leads to a rewiring of gene expression with reduced ribosomal capability, but increased lysosomal and catabolic activity. 4. Consistently with the gene signature, we found that SBDS loss causes a reduction in ATP and lactate levels, and increased susceptibility to DNA damage. Combining our data, we conclude that a cell-specific fragile phenotype occurs when SBDS protein drops below a threshold level, and propose a new interpretation of the disease.

## Author Summary

Shwachman Diamond syndrome (SDS) is an inherited disease. SDS presents, as hallmarks, exocrine pancreatic insufficiency, increased rate of infections, and higher incidence of leukemia. Most cases are due to mutations in the *SBDS* gene. *SBDS* encodes for a ribosome maturation factor. In this study, we immortalized mouse fibroblasts carrying one of the most common mutation of SDS patients and performed a thorough analysis of their properties. We show that the loss of SBDS activity causes a rewiring of gene expression and cellular metabolism. Overall we find a reduction of protein synthesis capability, a lower energy status, and increased lysosomal capability. SBDS mutant cells have an increased susceptibility to various forms of stress, but are strikingly resistant to oncogene-induced transformation. We propose a model that explains the complex phenotype of SDS patients and suggests roads for a rationale treatment.

## Introduction

Ribosomopathies are inherited diseases due to the haploinsufficiency of ribosomal proteins or ribosome processing factors [1, 2]. Patients affected by ribosomopathies present multiorgan phenotypes [2]. Some relatively common features are bone marrow and skeletal deficits, and cancer predisposition [3, 4]. At the cellular level, ribosomal haploinsufficiency may cause the induction of tumor suppressor p53 [2, 5]. Consequently, in some models the depletion of p53 reduces the deleterious effects of ribosomal haploinsufficiency [6–9] leading to the hypothesis that abnormal p53 is the pathogenic culprit. However, this is not true for all cases [10, 11].

Shwachman-Diamond Syndrome (SDS) is a recessive ribosomopathy affecting 1 in 76,000 births. SDS is a multisystem disorder presenting in the first year of life and characterized by the hallmark of exocrine pancreatic dysfunction. Another common symptom is the susceptibility to chronic infections accompanied by neutropenia [12, 13]. With variable penetrance, patients affected by SDS may have low stature, skeletal defects and cognitive impairment [14, 15]. Finally, high risk of acute myeloid leukemia (AML) is associated with older patients [16]. Loss-of-function mutations in the *SBDS* gene have been identified as the cause of the disease [17].

Several studies addressed the function of SBDS protein in mammals and of its yeast homolog. A concise survey will be presented. SBDS has a role in the maturation of 60S ribosomal subunits. Deletion of the yeast homolog *sdo1* is quasi-lethal, leading to pre-60S nuclear export defects. Importantly, point mutations of *tif6*, a gene necessary for 60S biogenesis [18], revert the quasi-lethal phenotype [19]. These and other studies, have led to a general model in which SBDS is necessary for the maturation of 60S subunits, in order to remove eIF6 (mammalian Tif6) from mature 60S subunits [20–22]. Since eIF6 controls 60S availability [23] and full translational activation [24, 25], manipulation of the binding of eIF6 to the 60S may be critical for restoring SBDS-mutant cells. A recent report suggested that SBDS contributes to the efficient translation of C/EBPα and C/EBP-β mRNAs, uORF-containing mRNAs that are, among others, indispensable regulators of granulocytic differentiation [26]. A general analysis of translated mRNAs depending from SBDS is still lacking. We do not know in detail whether other steps of 60S maturation beside eIF6 release are affected.

Several studies have attempted to pinpoint other functions of the SBDS protein, in the obvious effort to explain the multiorgan phenotype of patients. At the cellular level, several phenotypes associated to SBDS loss have been described. These phenotypes have been largely observed either in primary cells from patients, or upon shRNA experiments in cell lines. Increased apoptosis driven by FAS was seen in HeLa upon SBDS shRNA [27], and increased ROS production [28]. Increased apoptosis was also seen in SBDS-depleted HEK293 cells upon DNA and chemically induced endoplasmic reticulum stress [29], and in hemopoietic cells [30]. SBDS association with mitotic spindle has been proposed [31] and the lack of SBDS has been associated with genetic instability [32]. In general, reduced clonogenic potential is observed in hematopoietic precursors upon SBDS depletion [9, 33]. Reduced respiratory capability has been observed in mammalian and yeast cells lacking SBDS or its homolog, Sdo1p [34, 35]. Overall, it is unclear whether the cellular phenotypes ascribed to the loss of SBDS activity are direct or indirect, general or cell-specific, and have a relationship with protein synthesis.

Recently, a mouse strain in which one of the most frequent missense mutation found in SDS patients is modeled, R126T, has been produced [36]. This mouse model reproduces the clinical symptoms of SDS patients. We exploited the availability of this model to address the cell autonomous effects due to hypomorphic *SBDS* alleles. We have derived from these mice embryonic fibroblast cell lines, we have reconstituted control MEFs with wild type SBDS and then performed a full characterization of their properties. We addressed in *SBDS* mutant cells their predisposition to oncogenic transformation, changes in eIF6 binding, transcriptional and translational changes, metabolic parameters, and sensitivity to drugs and stresses. We unveiled several features due to the loss of SBDS. We provide a pathogenic model of SBDS deficiency that focuses on a diminished anabolic and energetic status of *Sbds* mutant cells due to reduced protein synthesis, which may be useful to design rational therapeutic ameliorative strategies.

## Results

### *Sbds* mutant cells are resistant to oncogene induced transformation

**Fig 1:**
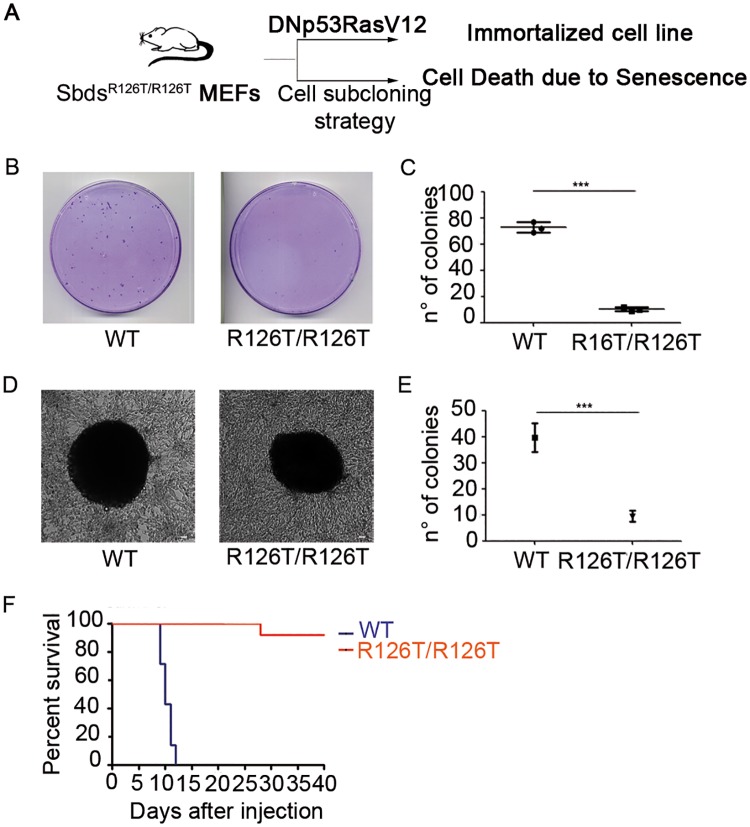
Cells with *Sbds*^*R126T/R126T*^ mutation show facilitated senescence, have reduced clonogenic potential, are refractory to oncogene induced transformation and generate tumor cells with poor growth *in vivo*. **(A)** Scheme of the strategy for immortalization from E12.5 MEFs from *Sbds*^*R126T/R126T*^ or wild type mice and summary data relative to *Sbds*^*R126T/R126T*^ cells. (**B-C)** Generation of immortalized colonies by infection of E12.5 MEFs at early passages with a retrovirus carrying DNp53 + H-ras^V12^. *Sbds*^*R126T/R126T*^ have low clonogenic potential. n = 3 per genotype). (B) representaive plates; (C) quantitation **(D-E)** Soft agar assay for transformed cells unveils that some *Sbds*^*R126T/R126T*^ transformants grow as well as wt (D), but overall they produce less colonies (E). Scale bar 25 μm. **(F)** *In vivo* tumor growth underscores an unexpected incapability of *Sbds*^*R126T/R126T*^ transformants to propagate tumors in mice. Kaplan-Meier curves of CD1 nude mice subcutaneously injected with *Sbds*^*R126T/R126T*^ and wild type MEFs (n = 7 per genotype): death time point is defined at 400mm^3^ tumor volume, when mice were euthanized. Number of colonies expressed as mean ± s.d. Statistic applied for **(B-E)** was t-test, paired, two-tailed (*P value≤0.05, ***P≤0.001). Log-rank (Mantel-Cox) Test (***P≤0.0001) was applied to (F).

Swhachman-Diamond (SDS) patients have an higher incidence of blood tumors, mainly acute myeloid leukemia (AML) [37]. This observation raises the question whether *SBDS* mutant cells are intrinsically susceptible to oncogene-mediated transformation. *SBDS* point mutation R126T corresponds to a common mutation found in SDS patients (c.377G>C) and it has been modeled in mice [36]. We immortalized R126T mouse embryonic fibroblasts (MEFs), together with their matched wild type controls, and we evaluated their capability to form colonies. Two strategies can be employed for immortalization of MEFs, a) sequential subcloning [38] or b) immortalization and transformation with oncogenes and tumor suppressor inhibitors [39]. By sequential subcloning, we were unable to derive *Sbds*^*R126T/R126T*^ MEFs due to early senescence (Fig 1A), whereas we normally derived wt cells. This result is in line with a recent work describing early senescence in the pancreas of *SBDS* mutants [8]. In contrast, immortalization of *Sbds*^*R126T/R126T*^ MEFs by infection with a vector carrying the dominant negative p53 and Ras G12V was successful (Fig 1A; S1A and S1B Fig). The growth of immortalized *Sbds*^*R126T/R126T*^ MEFs was virtually identical to the one of wt cells (S1C Fig). Transformation of immortalized cells is induced by long-term growth at confluency. Next, we analyzed the capability of immortalized *Sbds*^*R126T/R126T*^ MEF cells to form transformed colonies respect to wild type cells, upon long term culture at 100% confluency. We observed that *Sbds*^*R126T/R126T*^ MEFs formed less foci respect to the wild type cells (Fig 1B and 1C). We then plated wt MEFs and *Sbds*^*R126T/R126T*^ MEFs in soft agar, another indicator of transformation efficiency, to test their capability to grow in anchorage independent condition. We found that surviving clones of *Sbds*^*R126T/R126T*^ MEFs grew as well as wt cells (Fig 1D), but their overall number was lower than the one of wt MEFs (Fig 1E). In order to evaluate the capability of these transformed colonies to induce tumor *in vivo*, we injected 500.000 transformed cells in nude mice and monitored tumor mass growth. Surprisingly and strikingly (Fig 1F), formally transformed *Sbds*^*R126T/R126T*^ cells were inefficient (n = 1) or unable (n = 6) to grow *in vivo* respect to the wild type cells. We demonstrate that SBDS deficiency induces in a cell-autonomous fashion a growth and clonogenic deficit that can be unveiled when cells are challenged by environmental conditions.

### The primary effect of *Sbds* hypomorphism is an impairment in translation capability due to less mature 60S subunits

**Fig 2:**
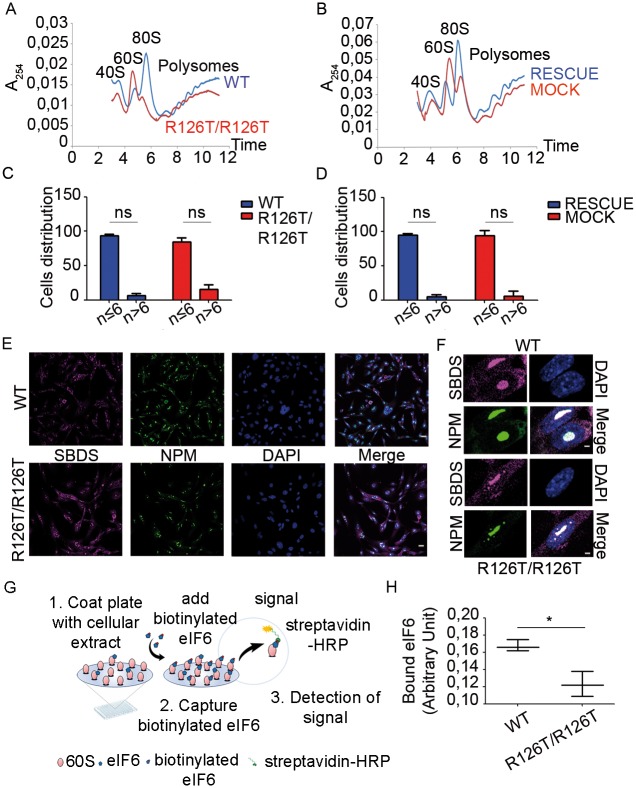
SBDS deficiency reduces the maximal translational capability up to 70% due to a defect in 60S maturation that is partly associated with a change in eIF6–free 60S subunits. **(A-B) Polysome profiles.** *Sbds*^*R126T/R126T*^ cells show an increase in free 60S and lower 80S peaks on sucrose gradient compared to wt (A). The phenotype is completely restored in *Sbds*^*RESCUE*^ cells (B). Note, the increase of free 60S is two-fold. A_254_ nm was measured after 15–50% sucrose gradient sedimentation. Representative experiment of n≥5. (**C-D) Nucleoli analysis.** *Sbds*^*R126T/R126T*^ (C) and *Sbds*^*MOCK*^ cells (D) do not have differences in the number of nucleoli respect to their control cell lines (wild type and *Sbds*^*RESCUE*^). Distribution of cells containing less or more of six nucleoli per nucleus in wild type, *Sbds*^*R126T/R126T*^_,_ *Sbds*^*RESCUE*^ and *Sbds*^*MOCK*^ cells was counted with Volocity Sofwtare, by analyzing cells stained for the nucleolar marker nucleophosmin (NPM) (n≥200, n = number of nuclei analyzed per genotype). **(E-F) SBDS and NPM localization.** Confocal images wild type and *Sbds*^*R126T/R126T*^ cells indicate a co-localization of SBDS and NPM proteins within the nucleolus, and a cytoplasmic SBDS. There are no visible differences among all genotypes. Scale bar 25 μm (E) and 2 μm (F). **(G-H)** Measurement of eIF6 binding sites by iRIA technique shows that wt cells have 25% more free 60S as detected by eIF6 binding. (G) iRIA technique outline: *Sbds*^*R126T/R126T*^ and wild type cellular extracts were immobilized on a 96 well and biotinylated eIF6 is added. This assay is able to detect the binding between eIF6 and 60S. (H) *Sbds*^*R126T/R126T*^ fibroblasts have less binding sites for eIF6, respect to wild type cell line, i.e. 0.12 arbitrary units versus 0.16. Representative technical triplicate experiment of n≥4 biological replicates.

We established a further model for studying SBDS function by generating, from immortalized *Sbds*^*R126T/R126T*^ MEFs, new clones retransduced with either wild type *Sbds* (*Sbds*^*RESCUE*^) or mock control (*Sbds*^*MOCK*^) vectors (S1D Fig). By comparing parental *Sbds*^*R126T/R126T*^ clones to their wild type counterparts, and the *Sbds*^*MOCK*^ with the *Sbds*^*RESCUE*^ clones, we can discriminate direct events due to SBDS lack from indirect effects. We describe the most important observations and discuss later a model that explains the pathogenic effect of SBDS deficiency. Since SBDS deficiency leads to a ribosomal defect [19], we performed a complete analysis of translation. Polysomal profiles can be used to analyze defects in initiation as well as in ribosome maturation. We observed, in line with previous reports [20], a strong unbalance in 60S and 80S peaks in *Sbds*^*R126T/R126T*^ MEFs respect to the wild type cells (Fig 2A). *Sbds*^*RESCUE*^ cells showed a complete rescue of the profile (Fig 2B), confirming a direct action of SBDS on ribosome maturation, and validating our model for discriminating direct versus clonal or indirect effects. We analyzed rRNA precursors with a pulse-chase assay by monitoring the incorporation of 5,6 ^3^H-uridine in the nascent ribosomal RNA (S2A Fig). We did not observe differences in rRNA maturation associated with the SBDS mutation, a result consistent with the idea that only the late maturation of 60S is affected by SBDS deficiency. The nucleolus is the nuclear compartment where both ribosome biogenesis and early maturation occur. A defect in ribosomal export can be assessed by measuring the number and the size of nucleoli. We did not observe differences in the number of nucleoli between *Sbds*^*R126T/R126T*^ and wild type cells (Fig 2C) or in *Sbds*^*MOCK*^ and *Sbds*^*RESCUE*^ cells (Fig 2D). In addition, we did not observe differences in co-localization of SBDS and nucleolar marker nucleophosmin (NPM) between *Sbds*^*R126T/R126T*^ and wild type cells (Fig 2E and 2F). eIF6 nucleolar localization was relatively similar in *Sbds*^*R126T/R126T*^ and wild type cells (S2B Fig).

eIF6 binds 60S subunits, blocking 80S formation and increasing free 60S peak [24]. It has been proposed that SBDS deficiency blocks eIF6 release [20]. This model is consistent with the accumulation of free 60S that we observed (Fig 2A). We recently developed a Ribosomes Interaction Assay (Fig 2G), able to quantify eIF6 binding sites on the 60S [40]. We immobilized equal ribosomes from *Sbds*^*R126T/R126T*^ and wild type cells, and measured eIF6 binding sites. We found a 25% reduction in eIF6 binding sites on the ribosomes of *Sbds*^*R126T/R126T*^, compared to wild type cells (Fig 2H). Thus, we conclude that SBDS deficiency leads to a late maturation deficit of 60S consistent with the generation of a reduced pool of functional 60S subunits. It is worth to note that 60S peak increases at least 2-fold (Fig 2A and 2B), whereas eIF6 binding sites drop only 25% (Fig 2H).

**Fig 3:**
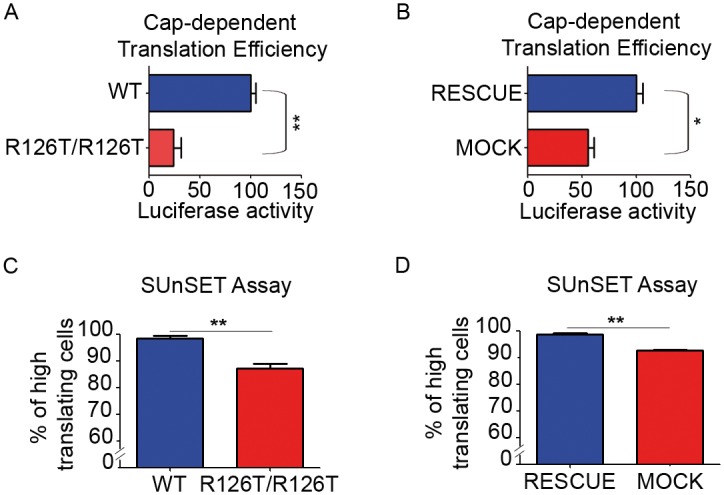
SBDS deficiency results in less translation capability. **(A-B)** *In Vitro* Translation Assay shows a 2- to 3-fold impairment in translation of SBDS-mutant extracts. Equal amounts of translation competent extracts prepared from indicated cells programmed with equal amounts of capped mRNAs. All graphs represent mean ± s.d. Statistic applied was T-test, paired, two-tailed, n≥4. **(C-D)** SUnSET single cell assay indicates a reduction in the number of cells incorporating mid-high puromycin both in *Sbds*^*R126T/R126T*^ (C) and in *Sbds*^*MOCK*^ fibroblasts (D) respect to their controls. Negative cells are low-expressing cells. Graphs represent mean ± s.d. Statistic applied was T-test, paired, two-tailed, n≥3.

We asked the consequences of the maturation deficit on translation. We developed a fully reconstituted *in vitro* model, in which translation competent extracts are prepared from equal amount of cells, normalized to the number of ribosomes and transduced with defined amounts of exogenous mRNA. This experiment allows to measure the maximal translational capability per cell/per ribosome. Ribosomal extracts from *Sbds*^*R126T/R126T*^ MEF showed around 70% reduction in the translational capability of a cap-dependent reporter (Fig 3A). Cells rescued with SBDS *in vivo* (*Sbds*^*RESCUE*^) recovered their translational capability (Fig 3B). Importantly, adding wild type SBDS *in vitro* did not rescue the translational capability of extracts prepared from *Sbds*^*R126T/R126T*^ MEFs (S2C Fig). This result indicates an overall translational impairment. Next, we adapted the canonical SUnSET protocol to citofluorimetry analysis. We observed both in *Sbds*^*R126T/R126T*^ MEFs respect to wild type (Fig 3C) and in *Sbds*^*MOCK*^ MEFs compared to *Sbds*^*RESCUE*^ MEFs (Fig 3D) about 10% reduction in the number of cells incorporating medium to high levels of puromycin. Taken together, our results demonstrate that R126T mutation leads to a strong reduction of the pool of 60S subunits competent for translation.

### SBDS deficiency causes limited changes in the translation of specific mRNAs but leads to snoRNAs’ accumulation on immature 60S

**Fig 4:**
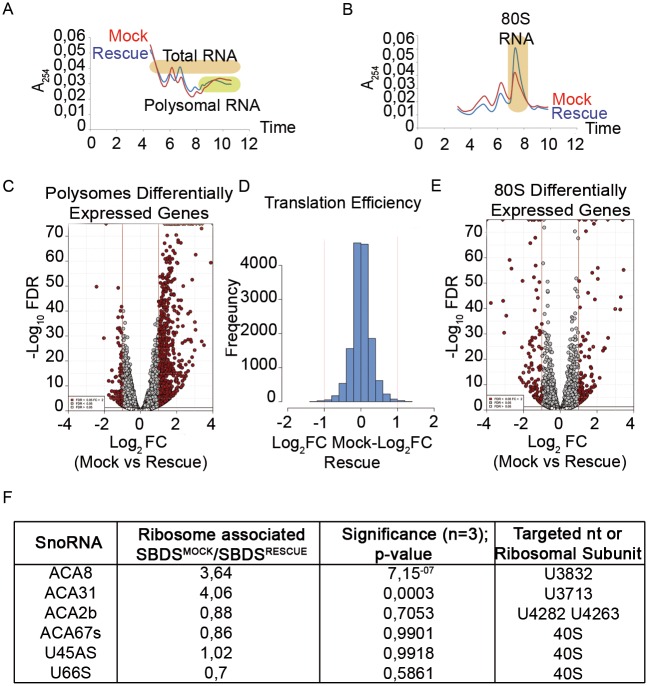
RNASeq of 80S and polysome associated RNAs shows that *SBDS* mutation causes limited changes in mRNAs associated to polysomes, accumulation of ACA8 and ACA31 snoRNAs on immature ribosomes. **(A-B)** Sucrose gradient separations for RNA extraction and RNAseq. In (A) RNA was extracted from polysomes (fractions corresponding to polysomes were collected in one polysome fraction) and from the whole gradient (100 μL from each fraction of the gradient were pulled in one total fraction). In (B) RNA was extracted from 80S and total (not graphed). Colored rectangles correspond to extracted fractions. **(C-D)** Translational analysis. *Sbds*^*MOCK*^ cells show 844 genes differently expressed genes on polysome fraction, most of them upregulated respect to the *Sbds*^*RESCUE*^ polysome fraction (C), but relative normalization to total (D) indicates that there is not a consistent change in the specific translational efficiency of individual mRNAs. (C) Volcano plot representing the differential expression analysis for polysome fraction. Results obtained comparing *Sbds*^*MOCK*^ versus *Sbds*^*RESCUE*^ conditions are reported for the pool of tested genes as -Log10 false discovery rate / Log2 moderate fold change. Genes selected as significantly changed (FDR < 5%, absolute Log2 fold-change value > 1) are shown in red. (D) Histogram showing the translational efficiency, calculated as delta Log2 fold-change (Polysome/Total) between *Sbds*^*MOCK*^ and *Sbds*^*RESCUE*^. The normal distribution indicates that very few genes are specifically regulated in the polysomal fraction, once calculated the Polysome/Total ratio. **(E)** Volcano plot representing the results of the differential expression analysis for the 80S fraction. **(F)** Sequencing unveils accumulation on ribosomes of *Sbds*^*MOCK*^ versus *Sbds*^*RESCUE*^ of some snoRNAs.

An obvious question is whether the impaired maturation of 60S ribosomes, associated with a reduced translational capability in *Sbds*^*R126T/R126T*^ cells, results in a qualitative difference of translation. We decided to proceed with an RNASeq study on total RNA extracted from sucrose gradient collected fractions. We studied 1) RNAs associated to polysomes, 2) RNAs associated to the 80S and 3) steady-state mRNA levels (total RNA) (Fig 4). The isolated fractions are shown in Fig 4A and 4B. The combination of these parameters allowed us to define the overall translational and transcriptional status associated with *Sbds*^*R126T/R126T*^ mutation, assuming that mRNAs differentially localized to either polysomes or 80S are controlled at the translational level. We have decided to use this strategy over ribosome profiling because a) altered 60S subunits may lead to abnormal RNA protection, b) the big change in the 80S monosomal peak found in *Sbds*^*R126T/R126T*^ cells is difficult to be normalized, and c) to efficiently reach deepness of more than 2x10^7^ reads. S1 File contains read counts of polysomes and total RNAs. S2 File contains read counts of 80S. By comparing the polysomes of *Sbds*^*RESCUE*^ and *Sbds*^*MOCK*^, we identified 844 modulated genes (Fig 4C; S1 File), most of them enriched in presence of mutant SBDS. However, when we estimated the translational efficiency, i.e. *bona fide* polysomal enrichment, by normalizing each mRNA level on the polysome to the amount present on the total, we found only 74 mRNAs with a significant modulation. Of these 74, 31 had less than 10 normalized read counts average expression, suggesting that fluctuations of poorly expressed mRNAs may contribute to the observed effects. The analysis of translation efficiency of *Sbds*^*RESCUE*^ and *Sbds*^*MOCK*^ confirmed that they do not differ in qualitative translational regulation (Fig 4D). The analysis on RNAs enriched on 80S subunits unveiled 250 genes modulated in 80S *Sbds*^*MOCK*^ compared to 80S from *Sbds*^*RESCUE*^ cells (Fig 4E; S2 File). However, identical to what we observed in the polysomal fraction, also in this case the mRNAs modulation on 80S followed the steady state levels (S3A and S3B Fig) as well the polysomal. Taken together, the data suggest that SBDS loss induces a solid transcriptional rewiring due to a general impairment of translation rather than specific translational regulation.

By analyzing polysomal versus steady state mRNAs and 80S modulated in *Sbds* mutated cells we made two major observations: a) a 4-fold enrichment of snoRNAs ACA8 and ACA31 in the ribosomes of *Sbds*^*MOCK*^ cells compared to *Sbds*^*RESCUE*^. ACA8 and ACA31 drive the pseudouridylation of 28S rRNA U3832 and U3713 on the 60S. Other snoRNAs were not changed (Fig 4F; S1 and S2 Files). Intriguingly, there was no overlap between the genes regulated at the polysomal level by *Sbds* mutation (S1 File), to the ones we previously found regulated by eIF6 deficiency [41].

In conclusion, our data (Figs 2G, 2H, 3 and 4) suggest that the lack of mature 60S leads to a general reduction of translational capability associated with transcriptional rewiring.

### The loss of functional SBDS causes an alteration in the transcriptome that indicates reduced synthetic and energetic capability but increased lysosome trafficking and altered proteostasis

**Fig 5:**
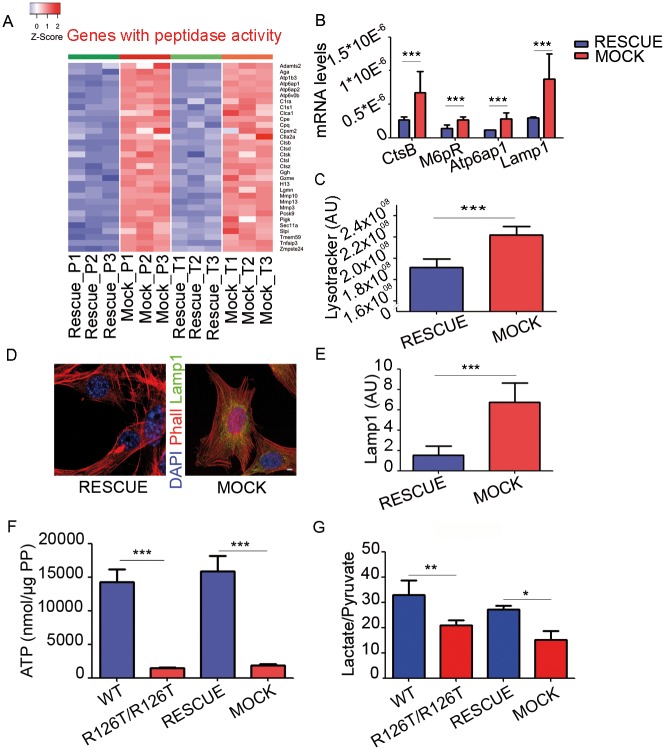
*SBDS* mutation results in increased lysomome trafficking and activity, and a decrease in ATP and lactate levels predicted by changes in the steady-state of the mRNAs identified by RNA-seq. **(A)** Changes in the steady-state levels (total, *bona fide* for transcriptional) of genes with peptidase activity as detected both on polysomes and total. Heat maps representing absolute gene expression levels in *Sbds*^*MOCK*^ and *Sbds*^*RESCUE*^ samples for the subset of gene sets with peptidase activity by Gene Ontology analysis. (**B)** qPCR validation of selected genes associated to lysosome activity. Real time analysis of selected genes associated to lysosome trafficking and activity. Data shown for *n*≥3; mean±s.d., T-test, paired, two-tailed. **(C)** *Sbds*^*MOCK*^ cells have a decrease in intracellular pH value. Representative graph showing increased lysotracker fluorescence intensitity in *Sbds*^*MOCK*^ cells respect to *Sbds*^*RESCUE*^ cells. This result suggests more lysosome trafficking and activity in *Sbds*^*R126T/R126T*^ cells. Data shown for *n*≥3; mean±s.d., T-test, paired, two-tailed. **(D-E)**. Lamp1 immunostaining (representative cells, D) and quantitation (E). Scale bar indicates 8.3μm. Globally, A-E show increase in the proteolitic potential of *Sbds*^*MOCK*^ respect to *Sbds*^*RESCUE*^ cells. **(F-G)** SBDS levels positively regulate ATP accumulation (F) and lactate/pyruvate production, an index of glycolysis (G). All graphs represent mean ± s.d. Statistic applied was T-test, paired, two-tailed, n≥4.

The limited specific translational changes seen at the polysomal level (Fig 4), and the strong reduction in the maximal translational capability (Fig 3) were mirrored by a complex rewiring of gene expression and metabolism of SBDS deficient cells (Fig 5). Hereafter, we describe the transcriptional and metabolic changes directly due to SBDS deficiency, i.e. fully rescued by SBDS readministration in the *Sbds*^*R126T/R126T*^ background. To simplify, 527 genes were at least 2-fold altered at the transcriptional level in *Sbds*^*MOCK*^ cells, all of them presenting concomitant changes in the polysomal pool. Functional analysis by classical Gene Ontology (GO) for the Molecular Function Domain was performed on these 527 genes. Twentyseven ontology terms grouped in ten emerging categories were found as significantly enriched. Gene Set Association Analysis (GSAA) on both polysomal and total fractions confirmed the findings of the classical (GO) gene ontology (S3 File). Globally, we found that SBDS mutant cells had a decrease in genes encoding for the ribosomal and respiratory chains, and an increase in the lysosomal capability. We will specifically describe some of them. We found upregulated (both at the polysomes and at the steady-state level) mRNAs with peptidase activity including lysosomal cathepsins such as *Ctsb*, *Ctsd*, *Ctsk* and *Ctsl* (Fig 5A), and lysosomal genes with vacuolar ATPase activity, including *Atp6ap1*, *Atp6ap2* and *Atpv0b* (Fig 5A, S1 File). Validation was confirmed by quantitative PCR (Fig 5B). *M6pr*, *Lamp1* and *Atp6ap1* upregulation suggests increased lysosomal activity associated with *Sbds* mutation, whereas increased *CtsB* suggests higher degradation activity. Consistently, we found in *Sbds*^*MOCK*^ cells an increase in cell acidification by the Lysotracker assay (Fig 5C) and an increase in Lamp1 positive cells by immunofluorescence (Fig 5D and 5E) and by western blot (S5A Fig). Other coordinated gene expression changes observed in *Sbds*^*MOCK*^ cells were a puzzling upregulation of membrane transporters for aminoacids and other intermediates (S4A Fig), a downregulation in most ribosomal components (S4B Fig), and a drop of expression of several genes important for mitochondrial function (S1 and S2 Files). These findings, in line with a reduction in the global protein synthesis capability observed in Fig 2G and 2H and in Fig 3, further predicted that *Sbds*^*R126T/R126T*^ cells might have a decrease in the level of high energy molecules like ATP. Therefore, we measured intracellular ATP levels and found a decrease in ATP levels both in *Sbds*^*R126T/R126T*^ and *Sbds*^*MOCK*^ cells respect to their controls (Fig 5F). The reduction of ATP levels was also confirmed in HEK cells carrying an shRNA for SBDS (S5B and S5C Fig). In addition, we did not observe any significant difference 1. AMPK and 2. phospoAMPK levels, whereas a mild difference was appreciate in 3. PhosphoAMPK substrates by western blots (S5D and S5E Fig). We then measured the levels of secreted lactate and pyruvate (S6A and S6B Fig), as index of the glycolytic flux, and we found a reduction in the Lactate/Pyruvate ratio both in *Sbds*^*R126T/R126T*^ and *Sbds*^*MOCK*^ respect to their controls (Fig 5G). To further understand the metabolism of these cells we measured the levels of a. glycolytic activity, b. respiration and c. ROS production. We found that both *Sbds*^*R126T/R126T*^ and *Sbds*^*MOCK*^ cells show a decrease in glycolysis (S6C Fig) and a reduction in respiration (S6D Fig), while ROS levels remained unchanged (S6E Fig). In conclusion, we demonstrate that the reduction of SBDS activity causes a reduction of a global translational capability and a cellular adaptation with less energy production and more compensatory catabolic capability.

We focused our attention on information deriving from LINCS/CMap project to evaluate if the gene expression profile found altered in the total fraction of *Sbds*^*R126T/R126T*^ MEFs cells was similar to transcriptional changes induced by drugs, observed in other cell types and to eIF6 deficiency [41]. Analysis performed by Query web tool on the complete list of modulated genes, and on a subset of the most changed ones identifies three common PKC activators: ingenol, phorbol-12-myristate-13-acetate and prostratin (S3 File).

### *Sbds*^*R126T/R126T*^ cells display a modest drug signature predicting stress sensitivity

**Fig 6:**
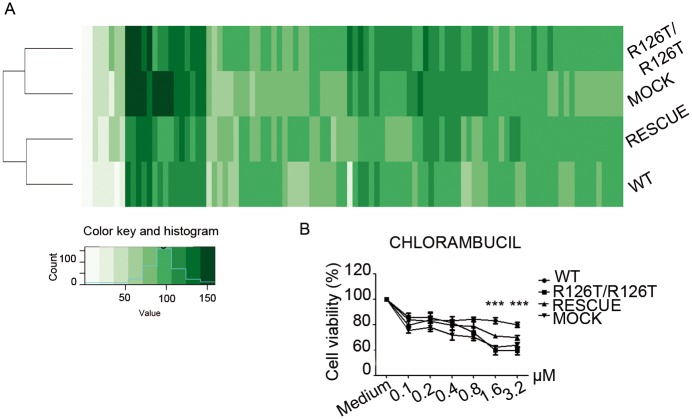
Drugs show a similar sensitivity profile between *SBDS* mutant or wt cells, but unveil a slight sensibility to selective DNA damage in *SBDS* mutant cells. **(A)** 100 molecules drug screening. Wild type, *Sbds*^*R126T/R126T*^_,_ *Sbds*^*RESCUE*^ and *Sbds*^*MOC*K^ MEFs were treated for 48h with 100 molecules selected from oral commercial drugs in UK. The heatmap shows a similar trend in all genotypes. However, the hierarchical clustering underlies that *Sbds*^*R126T/R126T*^ and *Sbds*^*MOCK*^ cells are more similar among them, than the wild type and *Sbds*^*RESCUE*^ cells. **(B)** Dose-response sensitivity to Chlorambucil a DNA alkylating drug. After the first large screening, molecules showing different activity in Sbds^R126T/R126T^ were selected for a second dose-response assay. The chemotherapic Chlorambucil is more toxic to *Sbds*^*R126T/R126T*^ and *Sbds*^*MOCK*^ MEFs (1,6 μM and 3,2 μM, 48h). Two-tailed t-test, paired (***P value P≤0.001).

Overall the analysis on SBDS deficient cells suggests that they have an impaired translational capability that leads to a compensatory transcriptional and metabolic rewiring that favors a catabolic processes and a state of low energy versus high synthetic capability. We reasoned that the transcriptional signature generated by SBDS deficiency could lead to a differential sensitivity to drugs or stressors which may be exploited at a therapeutic level. In principle, inhibitors that preferentially repress the growth of *Sbds*^*R126T/R126T*^ immortalized MEFs may be of use in treating SDS patients who are affected by Acute Myeloid Leukemia (AML). On the contrary, selective stimulators of growth could be potentially interesting for early-phase of the disease, for instance in the context of neutropenia or pancreatic insufficiency. We decided to proceed with two different screenings, the first based on 100 compounds selected from commercial oral drugs (S4 File). We evaluated the response signature to drugs associated to R126T mutation by measuring cell viability after 48h of treatment. We found that overall *Sbds*^*R126T/R126T*^ MEFs were similar to *Sbds*^*MOCK*^, and wt to *Sbds*^*RESCUE*^. This said, the signature profile that we found was surprisingly similar between wt and SBDS deficient cells (Fig 6A), with only one exception. In particular, we found increased sensitivity of *Sbds*^*R126T/R126T*^ and *Sbds*^*MOCK*^ cells to chlorambucil (Fig 6B), a DNA alkylating agent. Microtubule-targeting drugs mebendazole and colchicine were more toxic to *Sbds*^*R126T/R126T*^ cells (S7 Fig), instead niclosamide had a stimulatory effect on *Sbds*^*R126T/R126T*^ cells (S7 Fig), but the effects were not rescued in the *Sbds*^*RESCUE*^, suggesting indirect effects. The second screening with a Prestwick Library including 1280 compounds aimed at finding growth stimulators identified clindamycin (S7B Fig), but the effect was not rescued in *Sbds*^*RESCUE*^ cells. In conclusion, 1. *Sbds* mutation cause limited effects unless cells are challenged, 2. chlorambucil data predict that *Sbds*^*R126T/R126T*^ cells might be more sensitive to stress causing DNA damage.

**Fig 7:**
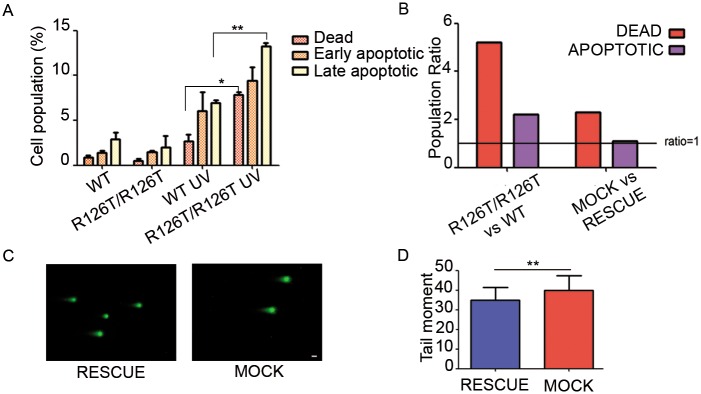
*Sbds*^*R126T/R126T*^ and *Sbds*^*MOCK*^ cells show an increase in cell death in response to UV stress. **(A-B)** UV exposure damages more SBDS mutant cells than wt or *Sbds*^*RESCUE*^. (A) Experiment on *Sbds*^*R126T/R126T*^ versus wt. There is an increase in cell death in *Sbds*^*R126T/R126T*^ versus wild type cells. In basal conditions no differences are observed among the genotypes (first two bar triplets, left). Upon UV damage cell death affects more *Sbds*^*R126T/R126T*^ (second two bar triplets, right). Cells were stressed with UV irradiation (9999 μJ/cm^2^, three times), recovered for 4 hours and stained for annexin with 7-AAD. **(B)** Observed ratios in all genotypes. Ratio ≥ 1 indicates higher sensitivity, the rescue with SBDS only partly restores the ratios. **(C-D)** COMET assay. Representative images of indicated cells treated as indicated in manufacturer protocol (Comet assay kit, Trevigen) in (C). Scale bars indicate 20 μm. (D) Absolute quantitation of the tail moment unveils a minor but significative difference.

The increased toxicity of the alkylating agent chlorambucil in *Sbds*^*R126T7R126T*^ cells hinted a possible increased susceptibility to cell death associated to other forms of DNA damage. We first measured the basal level of apoptosis in wild type and in *Sbds*^*R126T/R126T*^ cells by measuring the AnnexinV/7AAD positive population, and we did not find any differences between the two genotypes (Fig 7A). Treatment of cells with UV pulses caused an increase in cell death (Fig 7A), that was significantly more marked in *Sbds*^*R126T/R126T*^ and in *Sbds*^*MOCK*^ respect to wild type or *Sbds*^*RESCUE*^ cells (Fig 7B). We analyzed DNA damage by examining cells with the COMET assay (Fig 7C and 7D), where the tail moment revealed a slight but significant increase in DNA damage in *Sbds*^*MOCK*^ respect to wild type or *Sbds*^*RESCUE*^ cells. This effect, even if small, is consistent with increased sensitivity of *Sbds*^*MOCK*^ to two external stresses acting on DNA like chlorambucil and UV rays. These data, together with a decrease in energy levels and the uncapability to grow *in vivo*, show that *Sbds* mutated cells have an intrinsic fragility, that makes them weaker respect to their wild type counterpart.

## Discussion

**Fig 8:**
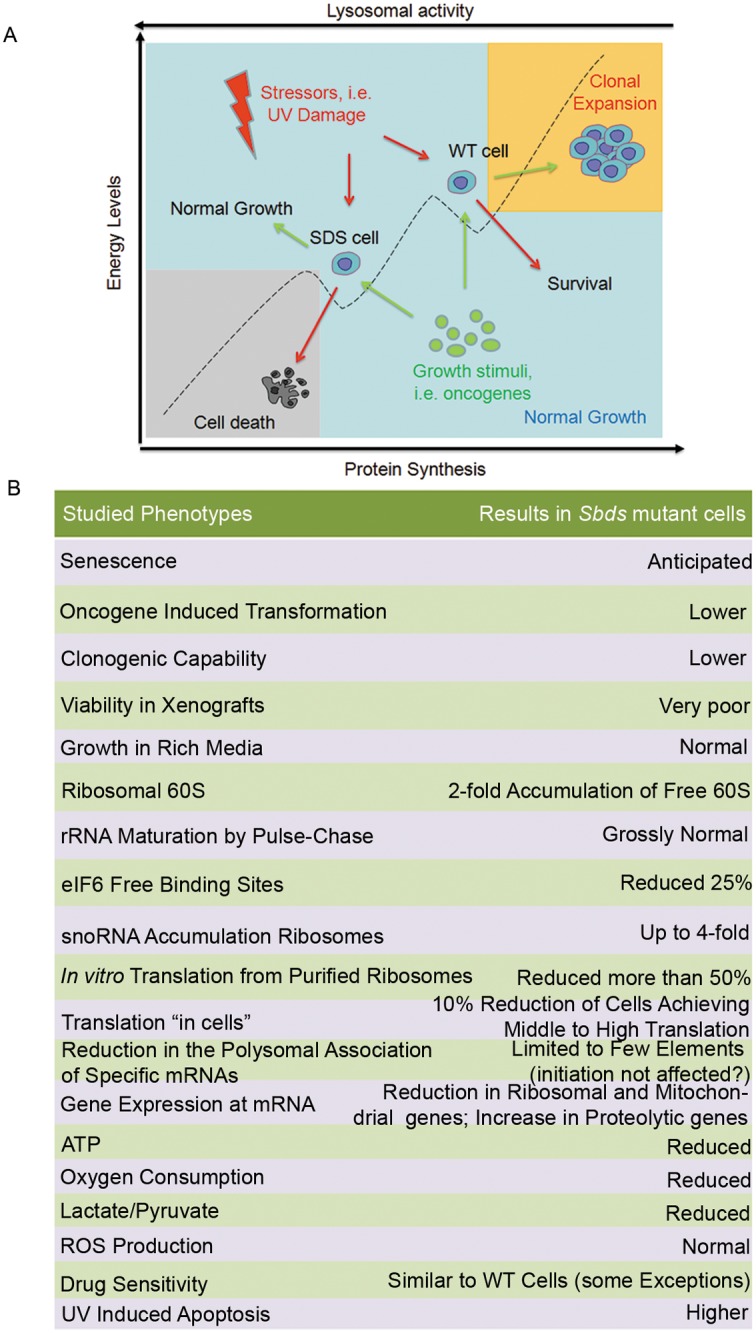
Model for SBDS-induced changes. **(A-B)** We speculate that SBDS mutation poises cells at a low energy level due to impaired global translation. Cells have a complex transcriptional rewiring and adapt by increasing the proteolytic flux. In this condition, they are less responsive to growth stimuli (example Fig 1), but more sensitive to stressors respect to wt cells. Interventions to increase translational efficiency are predicted to increase their fitness. **(B)** Summar table indicating all phenotypes observed in this study in *Sbds* mutant cells.

Our study suggests a model (Fig 8A) that may explain the pathogenic culprit of SDS and will be first described. The table in Fig 8B outlines the phenotypes that we have observed.

A simple model for SBDS-driven pathological symptoms is based on a threshold concept. Total null *SBDS* mutations are never observed in humans indicating that a zero threshold of SBDS is incompatible with life. Mutant SBDS has a residual activity. This activity is sufficient for, i.e., normal proliferation, but becomes limiting for efficient colony formation or escaping senescence. If senescence is bypassed by oncogene-driven immortalization and transformation, then, SBDS remains still limiting for survival in nude mice. We can therefore imagine that each individual cell, in each moment, may or may not meet the condition of insufficient SBDS activity, with subsequent effects. Indeed, by the SUnSET protocol we see that only some *SBDS* mutant cells do not reach high translation levels.

If the threshold is not reached, our study supports a model in which SBDS deficiency leads primarily to a delayed maturation of 60S ribosomal subunits. We confirm that the number of eIF6 binding sites on 60S ribosomes is reduced, consistent with reduced generation of free 60S and with a proposed model of SBDS-mediated eIF6 release in the late maturation of 60S, in cooperation with EFL1 [26]. In detail, we quantify a 25% decrease of eIF6 binding sites on 60S, similar to [42]. We also report that the increase of free 60S is 2-fold, the amount of accumulated ACA8/ACA31 snoRNAs on immature 60S is 4-fold, and ribosomal extracts from SBDS mutants have a 70% reduction in translational capability. Apparently, the ribosomal deficit due to SBDS mutation is more pervasive than expected and suggests the generation of a bulk of immature 60S that are poorly active in translation, even if eIF6 is released. This said, eIF6 release may be critical in the rescue of some mature ribosomes and the alleviation of pathological consequences.

A variety of proxyes due to reduced SBDS activity are evident. Indeed, adaptation is evident in the absence of overt signs of SBDS insufficiency. Even if SBDS mutant cells proliferate normally in rich media, they show a signature with reduced energy level, increased lysosomal capability, decreased ribosomal production and less oxygen consumption. The drug screening identified a subtle signature of fragility which may contribute to the pathology. Previously, it was reported excess protease secretion in SDS-derived iPSC [43], reduced respiration in yeasts and mammalian cell models [34], and less ATP [35] that together with our extended gene signature define a status of reduced anabolic capability. This may be a keyfactor for rescuing the phenotype. Stimulators of ATP production, for instance, may have an impact on the disease. Our initial drug screening suggests that efforts in this direction may be valuable: we screened drugs for survival activity and found that *SBDS* mutants are remarkably similar to wt cells. However, a screening for a closer proxy of SBDS deficiency such as ATP levels, may lead to successful compounds.

We think that increased AML in SDS patients is a defect due to impaired host-cancer interplay, rather than due to genetic instability. If anything, SBDS-deficient cells are very resistant to oncogenic transformation and unable to grow in unfavourable conditions like the ones generated in tumors (see xenografts). In the bone marrow of SDS models, the neutrophil lineage can be impaired by the absence of sufficient endogenous SBDS activity [9]. SDS patients have neutropenia [44]. It is known that severe congenital neutropenia is associated with the progression to acute myeloid leukemia [45]. Therefore, we speculate that neutropenia in the bone marrow niche [30, 42] may lead to expansion of tumor cells that cannot be eliminated by proper immunosurveillance. Similar suggestions came from mouse work [46]. In summary, for therapy, we need to address which cell is directly affected by SBDS deficiency.

A divergence between our studies and previously published works relates to ROS [28] production. In our hands we did not find evidence for increased ROS production. It is possible that impaired ROS balance is observed as an indirect effect driven by mitochondrial alterations in specific cells, but is not a direct proxy of SBDS deficiency.

Finally, we would like to comment on translational control driven by SBDS and eIF6. SBDS insufficiency generates a unique phenotype. Similarly to *SBDS* mutants, eIF6 depletion protects from oncogene-induced transformation [25, 47], whereas its amplification is oncogenic [48, 49]. eIF6 depletion does not induce senescence [25], but induces a different transcriptional rewiring [41], a divergent bone marrow phenotype affecting platelets [50], and a generally improved metabolic status [41]. SBDS depletion reduces the translation of mRNAs undergoing reinitiation, as CEBP/β derived LIP peptide [26]. In our study the decrease of CEBP/β mRNA on polysomes driven by SBDS deficiency is around 10% (S1 Table). eIF6 depletion reduces both reinitiation and LIP expression as well as the translation of G/C rich mRNAs [41]. To summarize, the specific effects of eIF6 loss and SBDS loss are different, but overall they suggest that both eIF6 and SBDS have a stimulatory role in protein synthesis. In yeast and *Dictyostelium* eIF6 mutants revert the SBDS loss [19, 20, 22]. We speculate that these mutants are gain-of-function genes and that eIF6 agonists may be beneficial to the disease.

In conclusion, our results support the idea that cells with reduced SBDS activity are able to grow and cycle as well as wild type cells in proper conditions, but if they drop below a SBDS threshold level they show a phenotype. However, the adaptation signature suggests logical roads for improving their fitness. In the long run a screening for compounds which may accelerate the release of eIF6 from 60S subunits can be helpful to discover specific drugs for treating SDS (assuming that a modest increase in active 60S can strongly ameliorate the phenotype). Alternatively, screening on proxyes such as ATP levels may lead to faster therapeutic options.

## Materials and Methods

### Cell culture and infections

Primary wild type and *Sbds*^*R126T/R126T*^ MEFs (E12.5) were grown in DMEM (Lonza), supplemented with 10% Fetal Bovine Serum (FBS) and 1% penicillin, streptomycin, L-glutamine, and maintained at 37°C and 9% CO_2_. Mycoplasma testing was performed before experiments. These cells were infected at early passages through retroviral vectors carrying DNp53 + oncogenic H-ras^V12^ [25]. After immortalization, cells were maintained at 37°C and 5% CO_2_. Immortalized *Sbds*^*R126T/R126T*^ MEFs were infected with lentiviral vectors carrying the wild type Sbds to generate the *Sbds*^*RESCUE*^ line or with the corresponding empty vector to obtain the *Sbds*^*MOCK*^ line. The lentiviral vectors (pHAGE-CMV-dsREDexpressIRESzsGreen backbone vectors) used to reconstitute SBDS wild type protein were kindly provided by A. Shimamura (Fred Hutchinson Cancer Research Center, Seattle). HEK-293T and HeLa cells from ATCC were cultured in DMEM (Euroclone) supplemented with 10% FBS and penicillin/streptomycin/glutamine solution (GIBCO) at 37°C and 5% CO_2_. Mycoplasma testing was performed before experiments. 293T cells were transfected at 60–70% confluence with pFCY, SBDS shRNA and pFCY scramble lentiviral vector (a generous gift from the lab of DC. Link, Washington University School of Medicine) to produce lentiviral particles. HeLa cells were infected with lentivirus at a confluence of 50%. Experiments were performed one week after infection. Silencing of protein was measured by western blot analysis. All animal experiments were carried out under the guidelines of the Canadian Council on Animal Care, with approval of procedures by The Animal Care Committee of the Toronto Centre for Phenogenomics, Toronto, AUP #0093.

### Transformation assay, soft agar and in vivo tumor growth

For the transformation analysis, primary fibroblasts were infected at early passage with DNp53 + H-ras^V12^ retroviral vectors and left to grow at overconfluency. Foci were counted 3 weeks after infection and cells were recovered for in vivo experiments. Eight-weeks old CD1 athymic nude mice were used for detecting tumor growth after a subcutaneous injection of in vitro transformed MEFs (500,000 cells/mouse). Tumor growth was monitored and animals were euthanized when the tumor reached the size of 400 mm3. All experiments involving mice were performed in accordance with Italian National Regulations. Experimental protocols were reviewed by local Institutional Animal Care and Use Committees. The soft agar formation assay and the focus formation assay were performed as described previously [51]. In vitro assays were performed at least three times, each in triplicate.

### Polysome profiles

Growing cells were lysed in 50 mM Tris-HCl, pH 7.5, 100 mM NaCl, 30 mM MgCl_2_, 0.1% Nonidet P-40, 100 μg/ml cycloheximide and 40 U/mL RNasin. After centrifugation at 12,000 r.p.m. for 10 min at 4°C, cytoplasmic extracts with equal amounts of RNA (10 OD_260_) were loaded on a 15–50% (or 10–30%) sucrose gradient dissolved in 50 mM NH_4_Cl, 50 mM Tris-Acetate, 12 mM MgCl_2_, 1 mM DTT and centrifuged at 4°C in a SW41Ti Beckman rotor for 3 h 30 min at 39,000 r.p.m. Absorbance at 254 nm was recorded by BioLogic LP software (BioRad) and fractions (1.5 mL each) were collected for subsequent proteins or RNA extraction. Each experiment has been performed at least six times, for each condition.

### RNA isolation and RNA sequencing and quantitative PCR

Total RNA was extracted from sucrose gradient aliquots. For the 15–50% gradient, we pulled fractions corresponding to polysomes in one fraction, and we pulled 100 μL from each fraction from the whole gradient in one fraction (total). For the 10–30% gradient, we pulled fractions corresponding only to the 80S peak in one fraction, named 80S, and we used as total RNA aliquots from the pre-gradient loading extract (pre-load). Afterward, we added to samples proteinase K (to a final concentration 100μg/mL) and SDS (to a final concentration of 1%) and we incubated them for 1 h at 37°C. Total RNA was then extracted by phenol/chloroform/isoamyilic acid method (https://tools.thermofisher.com/content/sfs/manuals/trizol_reagent.pdf). Libraries for Illumina sequencing were constructed from 100 ng of total RNA with the Illumina TruSeq RNA Sample Preparation Kit. The generated libraries were loaded on to the cBot (Illumina) for clustering on a HiSeq Flow Cell v3. The flow cell was then sequenced using a HiScanSQ (Illumina). A paired-end (2×101) run was performed using the SBS Kit v3 (Illumina). Experiments were performed in biological triplicates.

For quantitative PCR, 150 ng of RNA was retrotranscribed according to SuperScriptTM III First-Strand Synthesis SuperMix manufacturer protocol (18080400, Life Technologies). For RNASeq validation, Taqman probes specific for *Atp6ap1* (Mm01187488_g1), *Lamp1* (Mm00495262_m1), *Ctsb* (Mm01310506_m1) and *M6pr* (Mm04208409_gH) were used. Target mRNA quantification was performed by using ΔCt-method with 18S rRNA as an internal standard, performed on a StepOne Puls System (Applied Biosystems). Results are represented as means + s.d. of three independent experiments.

### Bioinformatics analysis

#### Read pre-processing and mapping

Three biological replicates were analyzed for polysomal, total and 80S fractions, with the exception of 80S *Sbds*^*RESCUE*^ condition, for which two biological replicates were available. Three replicates were examined for pre-load samples. Raw reads were checked for quality by FastQC software (version 0.11.2) (Available from: http://www.bioinformatics.babraham.ac.uk/projects/fastqc), and filtered to remove low quality calls by Trimmomatic (version 0.32) [52] using default parameters and specifying a minimum length of 50. Processed reads were then aligned to mouse genome assembly GRCm38 (Ensembl version 77) with STAR software (version 2.3.0e) [53].

#### Gene expression quantification and differential expression analysis

HTSeq-count algorithm (version 0.6.1, options = no, gene annotation release 77 from Ensembl) [54] was employed to produce read counts for each sample. To estimate differential expression, the matrix of read counts produced by HTSeq was analyzed by DESeq2 (version DESeq2_1.6.3) [55]. In a first step, differential expression analysis by DeSeq2 algorithm was performed to compare *Sbds*^*MOCK*^ versus *Sbds*^*RESCUE*^ genotypes in polysomal or in total fractions (12 samples overall). Read counts were normalized by calculating a size factor, as implemented in DESeq2. Independent filtering procedure was then applied, setting the threshold to the 65 percentile; 14471 genes were therefore tested for differential expression. Significantly modulated genes in polysomal or total fractions were selected by considering an absolute value of log2 fold change higher than 1 and a false discovery rate lower than 5%. In the same analysis framework, the moderate fold-change (as implemented by DeSeq2) was calculated for the polysomal versus the total fraction in *Sbds*^*MOCK*^ as well as *Sbds*^*RESCUE*^ conditions and considered as measure of the translational efficiency. The comparison between the two genotypes was obtained by calculating a delta Log_2_ fold-change between *Sbds*^*MOCK*^ and *Sbds*^*RESCUE*^ and visually represented by a histogram.

Regularized logarithmic (rlog) transformed values were employed for heat map representation of gene expression profiles.

Similarly, DESeq2 workflow was applied for the comparison of *Sbds*^*MOCK*^ versus *Sbds*^*RESCUE*^ in the 80S fraction (5 samples). Independent filtering threshold of 65% resulted in the selection of 14484 genes for statistical testing. In order to compare the levels of 80S and pre-load, DESeq2 normalization and regularized log transformation steps were performed on the samples belonging to these experimental conditions together, and the genes of interest were visualized by a heat map.

Analyses were performed in R version 3.1.2 (2014-10-31) (R Core Team (2015). R: A language and environment for statistical computing. R Foundation for Statistical Computing, Vienna, Austria. URL https://www.R-project.org/.).

#### Functional analysis by ClueGO

Cytoscape plug-in ClueGO (version 2.2.3) [56] was employed to perform gene ontology analysis and generate enrichment networks on the pool of 527 genes selected as modulated both in the polysomal and in the total fractions, using the group of 14471 tested genes as custom reference set. The Molecular Function domain of the Gene Ontology was considered. The following parameters were set: level 3–8, k = 0.4, Min Number of Genes = 8, Min Percentage = 8%, Pval < 0.01 (Enrichment; Bonferroni Step down).

### Gene set association analysis

Gene set association analysis for polysomal and total fractions was performed by GSAA software (version 1.2) [57]. Raw reads for about ~ 22000 genes identified by Entrez Gene ID were analyzed by GSAASeqSP, using gene set C5 (mouse version retrieved from http://bioinf.wehi.edu.au/software/MSigDB) and specifying as permutation type ‘gene set’ and as gene set size filtering min 15 and max 800.

### CMap analysis

Analysis on CMap/LINCS gene signatures was performed using the ‘Query’ web tool (http://apps.lincscloud.org/query), that, taking as input a list of genes, computes the connectivity between this set and the gene expression signatures of the LINCS database. Human orthologs of mouse genes were retrieved relying on Biomart web tool (http://www.ensembl.org/biomart); only genes with a one-to-one orthologs relationship were maintained for downstream computations. Query tool was first applied on the list of 726 mouse genes significantly modulated in the total fraction; chemical compounds showing a mean rank value of at least 90 on 6 cell lines were selected as interesting. As second strategy, the same procedure was applied on the genes showing a robust modulation (143 genes with a fold-change higher than 3 as absolute value) and then selecting chemical compounds with a mean rank value of at least 90 on 4 cell lines. To find perturbations that are consistently retrieved, the overlap between the two analyses was considered as final result.

### Western blotting and antibodies

SDS-PAGEs were performed on protein extracts obtained with RIPA buffer (10 mM Tris-HCl, pH 7.4, 1% sodium deoxycholate, 1% TritonX-100, 0.1% SDS, 150 mM NaCl and 1 mM EDTA, pH 8.0). Protein concentration was determined with BCA analysis (Thermo Fisher Scientific). Equal amounts of proteins were loaded on each lane and separated on a 10% SDS-PAGE, then transferred on a PVDF membrane. The membranes were blocked in 10% Bovine Serum Albumin (BSA) in Phosphate Buffer Saline (Na_2_HPO_4_ 10 mM, KH_2_PO_4_ 1.8 mM, NaCl 137 mM, KCl 2.7 mM, pH 7.4) (PBS) with Tween (0,01%) for 30 minutes at 37°C. The following primary antibodies were used: β-actin (CST 4967L 1:4000), SBDS (Santa Cruz S15 SC49257 1:500) H-RAS (Santa Cruz SC520, 1:1000), Lamp1 (Santa Cruz SC20011, 1:200), AMPK (CST 5831 1:1000), phospho-AMPK (CST 2535 1:1000), phospho-AMPK Substrates (CST 5759, 1:1000). The following secondary antibodies were used: donkey anti-goat IgG HRP (Santa Cruz SC2020, 1:2000), donkey anti-rabbit IgG HRP (Amersham NA934 1:5000) and donkey anti-mouse IgG HRP (Santa Cruz, SC2005 1:5000). Each experiment was performed at least three times, each time in triplicate.

### Immunofluorescences and antibodies

Cells were seeded the day before the staining. The next day, cells were rinsed three times with PBS then fixed with ice cold methanol 100% for 10 minutes at -20°C. After three washes with PBS, cells were blocked in Normal Goat Serum 5%, for 1 hour at room temperature. Primary antibodies were incubated overnight at 4°C, and after three washes with PBS, secondary antibodies were incubated 3h at room temperature in the dark. After three washes with PBS, cells were incubated with DAPI (Molecular Probes NucBlue Live ReadyProbes Reagent R37605) as manufacturer protocol, then washed and mounted on slides with Mowiol 20%. All the antibodies were diluted in blocking solution. The following primary antibodies were used: NPM [25], SBDS (Santa Cruz S15 SC49257 1:25), eIF6 [58] (1:100), Lamp1 (Santa Cruz sc-20011 1:100). The following secondary antibodies were used: donkey anti-goat, donkey anti-mouse, donkey anti-rabbit (Alexa Fluor secondary antibodies, Molecular Probes 1:500). The cells were examined by confocal microscopy (Leica SP5) and analyzed with Volocity 6.3 software (Perkin Elmer). Immunofluorescence experiments were performed at least three times, in triplicate.

### Comet assay

Comet assay was performed following the manufacturer protocol (Trevigen, 4250-050-K). The stained nuclei were then examined by confocal microscopy (Leica SP-5), and analyzed (n≥30 for each condition) with Comet Assay IV software (Perceptive Instruments). The experiment was performed three times in triplicate.

### Ribosome biogenesis

Ribosome biogenesis was analyzed by pulse-chase experiments by adding 5,6-^3^H-Uridine to the medium (final concentration 3uCi/mL, NET367001MC PerkinElmer). 5,6-^3^H -Uridine was removed after 0, 10, 20 and 40 minutes of incubation and RNA was extracted with Trizol Reagent and hybridized on nitrocellulose membrane. This experiment was reproduced twice.

### In vitro translation assay

For *in vitro* translation assays, we used cell extracts prepared as described [59] with some optimizations. 80% confluent cells were trypsinized and lysed for 45 minutes at 4°C in 10 mM HEPES pH 7.6, 10 mM potassium acetate, 0.5 mM magnesium acetate, 5 mM DTT and protease inhibitor (Promega). Lysates were homogenized by syringing through a 27G, 3/4-inch needle. Lysates were clarified by centrifugation at 18.000 g for 1 minute and protein concentration was determined by BCA quantification assay. To make translation fully dependent on exogenously added mRNA, lysates were treated with 15 U/ml Micrococcal nuclease (MN Boehringer) and 0.75 mM CaCl_2_ and incubated at 25°C for 7 minutes. EGTA was added to terminate the reaction.

For the translation assay, 6 μl of cell extract were mixed to 1.2 μl of Master Mix (125 mM HEPES, 10 mM ATP, 2 mM GTP, 200 mM creatine phosphate, 0.2 mM aminoacid mix without methionine (Promega), 0.25 mM spermidine, 20 mM of L-methionine, 50 mM potassium acetate, 2.5 mM magnesium acetate, 20 U of RNAsin (from Promega) and 0.5 μg of purified reporter-encoding mRNA). The mRNA was obtained by using the Megascript T7 kit (Ambion) to perform the in vitro transcription reaction supplemented with 2 mM cap analog [M7G(5')PPP(5')G] (Ambion). The mix for the in vitro translation reaction was then incubated for 90 minutes at 30°C. Dual-Glo Luciferase Assay kit (Promega) was used to read Firefly and Renilla luciferase output with GloMax Luminometer (Promega). In vitro translation experiments were performed at least three times, in triplicate.

### SUnSET assay

For protein synthesis measurements, we adapted SUnSET protocol [60]. 70% confluent cells were treated with 5μg/ml puromycin for 10 minutes, then trypsinized and centrifugated 5 minutes at 500 g. Then, cell were fixed and permeabilized using reagents from the Foxp3/Transcription Factor Staining Buffer Set (Affymetrix, 5523–00), incubated with anti-puromycin 12D10 (1:2000, Millipore, MABE343) and finally with secondary antibody Alexa Fluor 488 Goat anti-Mouse (Invitrogen) and DAPI, according to manufacturer protocol. Cells were then analyzed with FACSCanto II (BD Bioscience) and analyzed with FlowJo8.8.7 software. SUnSET experiments were performed at least three times, in triplicate.

### Drug screenings

#### 100 molecules screening

Cells were plated in 96 well (6000 cells/well) 4 hours before drug treatments. The list of all drugs with their relative concentration is in the S4 File. Unless specified, the drug treatment time is 48 hours. Cell viability was measured with Cell Titer Blue (Promega, G8080), according to the manufacturer’s protocol. All treatments were performed at least three times, in triplicate.

### FDA screening

1280 approved drugs were analyzed for their impact on Sbds^*R126T/R126T*^ cells growth. Cells were seeded using a Multidrop Combi (Thermo Scientific) in 384 well plates (Perkin Elmer Cell Carrier, collagen coated) at a density of 4000 cells/well. After 24 hours the drugs were supplemented using a Hamilton Starlet liquid handler at a final concentration of 10 μM and cells were treated for 30 hours. The reference compound for toxicity was 5 μM Trichostatin A. Cytotoxicity was than evaluated by measuring in vivo nuclear shrinkage and loss of cells using Hoechst33342, and cell membrane disruption using the cell-impermeant nuclear dye BOBO-3. Briefly, following incubation with the drugs diluted in 50 μl growth medium, 25 μl of a dye cocktail containing 3 μM Hoechst33342 and 2.25 μM BOBO-3 were added and incubation was continued for further 45 min at 37°C, 5% CO_2_. Images were then immediately acquired and analyzed using the Operetta microscope (Perkin Elmer). For the validation of hits, 6-point dose-responses for each compound in duplicate were used (ranging from 30uM to 0.12uM). The number of dead cells/total number of cells was the readout of the assay, where dead cells correspond to nuclei stained with BOBO-3 and the total number of cells correspond to the nuclei stained with Hoechst33342.

### Metabolism analysis

#### Secreted lactate

Cells were plated in 6 well dishes in high-glucose medium for 24 hr and then switched to serum-free high-glucose (4,5 g/L) for 4 hr as indicated in [61]. Lactate secreted into the medium was measured using a fluorogenic assay, Lactate Assay Kit (Biovision). Average of fluorescence intensity was calculated for each condition replicates. Values were normalized to protein content obtained from the same wells.

#### ATP levels

For ATP measurements, samples were homogenized in ice-cold ATP buffer (20 mM Tris, pH 7.5, 0.5% Nonidet P-40, 25 mM NaCl, 2.5 mM EDTA) for 5 min. Lysates were centrifuged at 13,000 g for 30 min. Proteins were quantified by BCA analysis. Luminometric determination of ATP was assayed using the ATP-determination kit (Molecular Probes) according to [62].

#### Glycolysis and extracellular O_2_ consumption assays

For respiration and glycolysis analysis, fluorogenic kits were used (respectively, Abcam ab197243 and ab197244), following manufacturer protocols. Average of fluorescence intensity was calculated for each replicate, and then values were normalized for cells number.

#### Pyruvate levels

For pyruvate analysis, a fluorimetric kit was used (BioVision, K609-100) following manufacturer protocol. Average of fluorescence intensity was calculated for each replicate, and then values were normalized for protein content obtained. All assays were performed at least three times, in triplicates.

### Cell death and ROS production measurement

All analysis were performed using the FACSCanto II Flow cytometer (BD) and analyzed with FlowJo software (BD). All experiments were performed when cells reached the confluence of 70%. For the apoptosis analysis, cells were UV irradiated with a cross-linker (9999 μJ/cm2, three times) and cell death was detected by using the 7AAD-Annexin V kit (640926, BioLegend). For ROS measurement, cells were incubated with 50 μM H2DCF-DA (2’, 7’, -dichlorodihydrofluorescein diacetate, AbCam Ab113851) for 30 min at 37°C and 5% CO2 and immediately analyzed by flow citometry. All experiments were performed at least three times, in triplicate.

### In vitro ribosome interaction assay (iRIA)

iRIA assay was performed as described in [40]. Briefly, 96-well plates were coated with a cellular extract diluted in 50 μL of PBS, 0,01% Tween-20, O/N at 4°C in humid chamber. Coating solution was removed and aspecific sites were blocked with 10% BSA, dissolved in PBS, 0,01% Tween-20 for 30 minutes at 37°C. Plates were washed with 100 μl /well with PBS-Tween. 0,5 μg of recombinant biotynilated eIF6 was resuspended in a reaction mix: 2,5 ;mM MgCl_2_, 2% DMSO and PBS-0.01% Tween, to reach 50 ;μl of final volume/well, added to the well and incubated with coated ribosomes for 1 hour at room temperature. To remove unbound proteins, each well was washed 3 times with PBS, 0,01% Tween-20. HRP-conjugated streptavidine was diluted 1:7000 in PBS, 0,01% Tween-20 and incubated in the well, 30 minutes at room temperature, in a final volume of 50 μl. Excess of streptavidine was removed through three washes with PBS-Tween. OPD (o-phenylenediamine dihydrochloride) was used according to the manufacturer’s protocol (Sigma-Aldrich) as a soluble substrate for the detection of streptavidine peroxidase activity. The signal was detected after the incubation, plates were read at 450 nm on a multiwell plate reader (Microplate Bio-Rad model 680). This experiment was performed at least three times, in triplicate.

### Lysotracker assay

Cells were seeded the day before the assay. The next day, cells were incubated with Lysotracker DeepRed (Thermo Fisher Scientific L12492) according to the manufacturer protocol. The cells were examined with Nikon Ti-Eclipse microscope and analyzed with Volocity 6.3 software (Perkin Elmer). For subsequent analysis, cells were then fixed with paraformaldehyde 3% for 10 minutes at room temperature and nuclei were stained with DAPI (Molecular Probes NucBlue Live ReadyProbes Reagent R37605) as manufacturer protocol, then washed and mounted on slides with Mowiol 20%. The cells were examined by confocal microscopy (Leica SP5) and analyzed with Volocity 6.3 software (Perkin Elmer). Lysotracker experiments were performed at least three times, in triplicate.

### Accession number

The RNA seq data are available at www.ebi.ac.uk/arrayexpress with accession number ID: E-MTAB-5089.

## Supporting Information

S1 Fig Normal features of immortalized *Sbds*^*R126T/R126T*^ MEFs. **(A)** Morphological appearance of immortalized *Sbds*^*R126T/R126T*^ MEFs. Representative phase-contrast picture of the immortalized cell line obtained with DNp53 + H-rasV12 oncogenes infection. Scale bar indicates 25 μm **(B)** Ras overexpression. Representative Western Blot of not-infected or DNp53 + H-ras^V12^ infected cells indicating the overexpression of *ras* oncogene. The blot was performed one week after the infection. **(C)** Doubling Time. Doubling time of multiple immortalized WT and *Sbds*^*R126T/R126T*^ MEFs. The doubling time was measured with a cell viability assay, at three different time points (24h, 48h, 72h). **(D)** SBDS protein levels. Representative Western Blot on wt, *Sbds*^*R126T/R126T*^, *Sbds*^*RESCUE*^ and *Sbds*^*MOCK*^ fibroblasts shows increased levels of SBDS protein in *Sbds*^*RESCUE*^ MEFs after infection with a lentiviral vector carrying the wild type form of SBDS. The blot was performed one week after the infection.
(TIF)

S2 Fig *Sbds*^*R126T/R126T*^ mutation is not associated to alteration in rRNA precursor, but leads to an impairment in CAP-dependent translation that is not rescued after the addition of SBDS peptide. **(A**) Pulse and chase assay. Ribosomal RNAs precursors were analyzed with 5,6^3^H-Uridine incorporation in *Sbds*^*R126T/R126T*^_,_ *Sbds*^*RESCUE*^ and *Sbds*^*MOCK*^ MEFs at four different time points (0, 10, 20 and 40 minutes of incubation with medium supplemented with 3 μCi/mL ^3^H-Uridine). There are no differences among genotypes analyzed. **(B)** SBDS and eIF6 localization. Confocal images on wild type and *Sbds*^*R126T/R126T*^ cells indicate the same co-localization of SBDS and eIF6 proteins within the nucleus. Scale bar 25 μm. **(C)** In Vitro Translation Assay. Luciferase activity was measured as index of CAP-dependent translation efficiency and indicates that *Sbds*^*R126T/R126T*^ fibroblasts have less capability respect to wild type cells. No rescue has been observed by adding the wild type SBDS protein. Graphs represent the mean of values, error bars indicates standard deviation. Two-tailed t-test, paired (*P value≤0.05, ***P≤0.001).
(TIF)

S3 Fig Differentially expressed genes in polysome and in 80S fractions are mostly shared by their total fractions. **(A-B)** Heat maps representing gene expression profile in polysomal and total fractions (biological replicates) for the pool of 844 genes identified as significantly changed in polysomes **(A)** and in 80S **(B)** and pre-load samples for the pool of 250 genes selected as significantly changed in 80S fraction. Values are represented as z-scores after rlog transformation.
(TIF)

S4 Fig Differentially expressed genes in total mRNAs. **(A-B)** Heat maps representing gene expression profile of mRNAs from the solute carrier genes class (A) and for the structural constituent of the ribosome (B). Values are represented as z-scores after rlog transformation.
(TIF)

S5 Fig Lamp1, ATP and phosphoAMPK/AMPK/AMPK substrates levels. **(A)** Representative Western Blot showing increased levels of Lamp1 in *Sbds*^*MOCK*^ cells respect to *Sbds*^*RESCUE*^ MEFs. **(B)** Representative Western Blot showing the levels of SBDS protein in HEK-293T cells infected with the pFCY SBDS shRNA lentiviral vector (SBDS shRNA) or pFCY scramble vector (scramble). **(C)** ATP levels in SBDS shRNA HEK-293T cells were reduced when compared to control cells. Graphs represent the mean of values, error bars indicates standard deviation. Two-tailed t-test, paired (*P value≤0.05, ***P≤0.001). **(D)** Representative Western Blot showing that wt, *Sbds*^*R126T/R126T*^, *Sbds*^*RESCUE*^ and *Sbds*^*MOCK*^ MEFs have the same levels of AMPK and phosphoAMPK proteins. **(E)** Representative Western Blot showing that both *Sbds*^*R126T/R126T*^ and *Sbds*^*MOCK*^ MEFs have a mild increase in phosphoAMPK substrates compared to wild type and *Sbds*^*RESCUE*^ cells.
(TIF)

S6 Fig SBDS deficiency is associated to altered metabolism. *Sbds*^*R126T/R126T*^ and *Sbds*^*MOCK*^ MEFs display a significant decrease in lactate **(A)** and pyruvate **(B)** levels, also confirmed by a reduction in glycolytic activity **(C)**, measured with extracellular pH levels, respect to their controls wild type and *Sbds*^*RESCUE*^ MEFs. *Sbds*^*R126T/R126T*^ and *Sbds*^*MOCK*^ MEFs display also a reduction in respiration **(D)**, but ROS levels **(E)** remain unchanged. Graphs represent the mean of values, error bars indicates standard deviation. Two-tailed t-test, paired (*P value≤0.05, ***P≤0.001).
(TIF)

S7 Fig Differential sensitivity to drugs observed in the *Sbds*^*R126T/R126T*^ versus wt, but not recovered in the *Sbds*^*RESCUE*^. After the first large screening, molecules showing different activity in *Sbds*^*R126T/R126T*^ have been selected for a second dose-response assay including the *Sbds*^*RESCUE*^. Four drugs confirmed the data on the parental cell lines but were not rescued, suggesting clonal variations or indirect effects. **(A)** Niclosamide. **(B)** Clindamycin. **(C)** Mebendazole. **(D)** Colchicine.
(TIF)

S1 File Complete read counts of polysomal and total fraction. List of all genes detected and tested for differential expression analysis in polysomal and total fraction for *Sbds*^*MOCK*^ and *Sbds*^*RESCUE*^. Gene quantification is calculated as normalized read counts.
(XLSX)

S2 File Complete read counts of polysomal and total fraction. List of all genes detected and tested for differential expression analysis in 80S fraction for *Sbds*^*MOCK*^ and *Sbds*^*RESCUE*^. Gene quantification is calculated as normalized read counts.
(XLSX)

S3 File Functional analysis of translational and transcriptional changes associated to R126T mutation. Gene Ontology annotation results; GSAA analysis on polysome and total fractions; LINCS/CMap analysis results.
(XLSX)

S4 File 100 molecules drug screening. Concentration of 100 molecules used in the 100 molecules drug screening with relative effects on all genotypes tested.
(XLSX)
