# Supplementary figures and images for: SBDS-Deficient Cells Have an Altered Homeostatic Equilibrium due to Translational Inefficiency Which Explains their Reduced Fitness and Provides a Logical Framework for Intervention
Source: PLoS Genet. 2017 Jan 5;13(1):e1006552. doi: 10.1371/journal.pgen.1006552 (PMC5249248; doi:10.1371/journal.pgen.1006552)

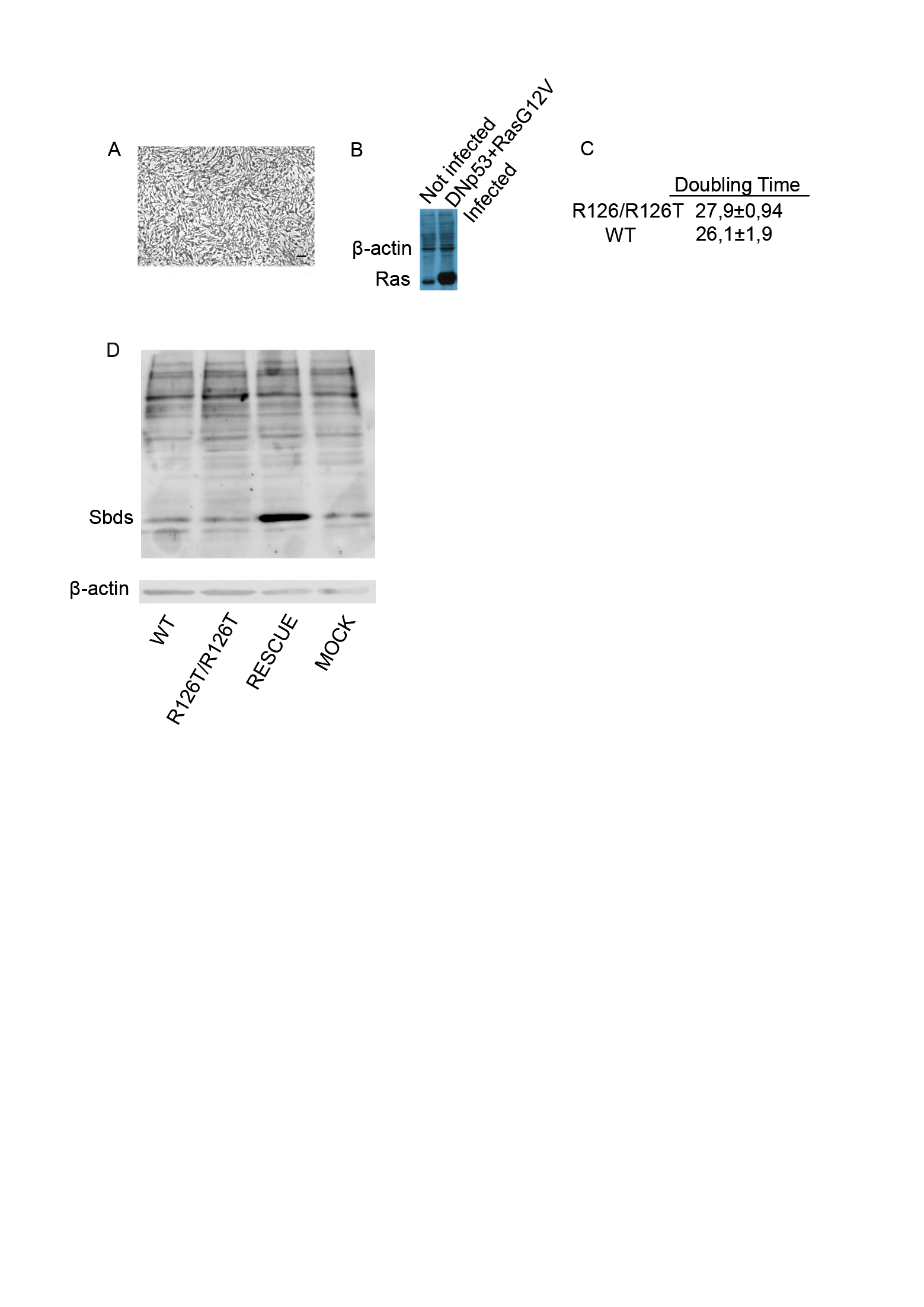

Supplement: S1 Fig — (A) Morphological appearance of immortalized SbdsR126T/R126T MEFs. Representative phase-contrast picture of the immortalized cell line obtained with DNp53 + H-rasV12 oncogenes infection. Scale bar indicates 25 μm (B) Ras overexpression. Representative Western Blot of not-infected or DNp53 + H-rasV12 infected cells indicating the overexpression of ras oncogene. The blot was performed one week after the infection. (C) Doubling Time. Doubling time of multiple immortalized WT and SbdsR126T/R126T MEFs. The doubling time was measured with a cell viability assay, at three different time points (24h, 48h, 72h). (D) SBDS protein levels. Representative Western Blot on wt, SbdsR126T/R126T, SbdsRESCUE and SbdsMOCK fibroblasts shows increased levels of SBDS protein in SbdsRESCUE MEFs after infection with a lentiviral vector carrying the wild type form of SBDS. The blot was performed one week after the infection. (TIF) [file pgen.1006552.s001.tif]

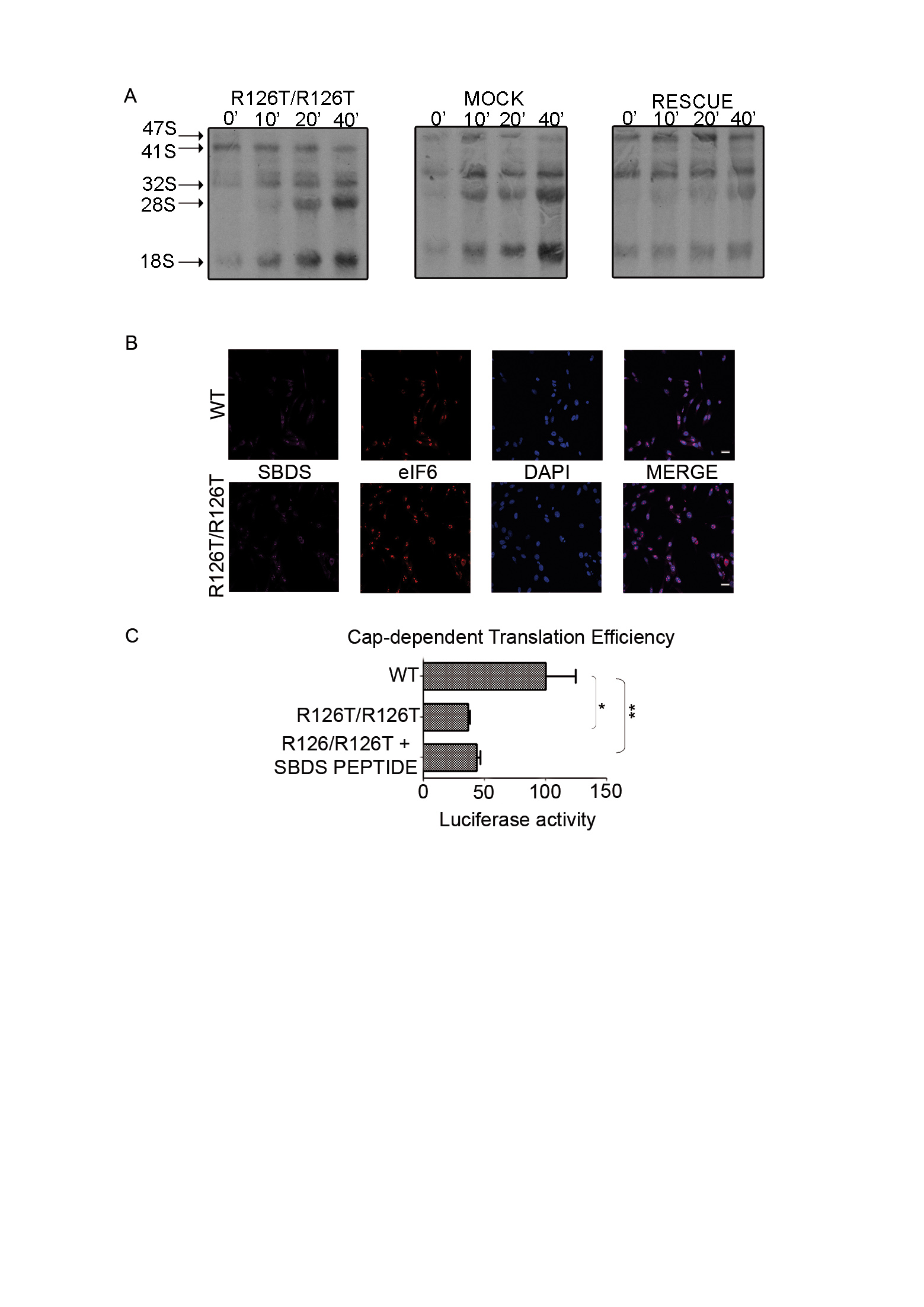

Supplement: S2 Fig — (A) Pulse and chase assay. Ribosomal RNAs precursors were analyzed with 5,63H-Uridine incorporation in SbdsR126T/R126T, SbdsRESCUE and SbdsMOCK MEFs at four different time points (0, 10, 20 and 40 minutes of incubation with medium supplemented with 3 μCi/mL 3H-Uridine). There are no differences among genotypes analyzed. (B) SBDS and eIF6 localization. Confocal images on wild type and SbdsR126T/R126T cells indicate the same co-localization of SBDS and eIF6 proteins within the nucleus. Scale bar 25 μm. (C) In Vitro Translation Assay. Luciferase activity was measured as index of CAP-dependent translation efficiency and indicates that SbdsR126T/R126T fibroblasts have less capability respect to wild type cells. No rescue has been observed by adding the wild type SBDS protein. Graphs represent the mean of values, error bars indicates standard deviation. Two-tailed t-test, paired (*P value≤0.05, ***P≤0.001). (TIF) [file pgen.1006552.s002.tif]

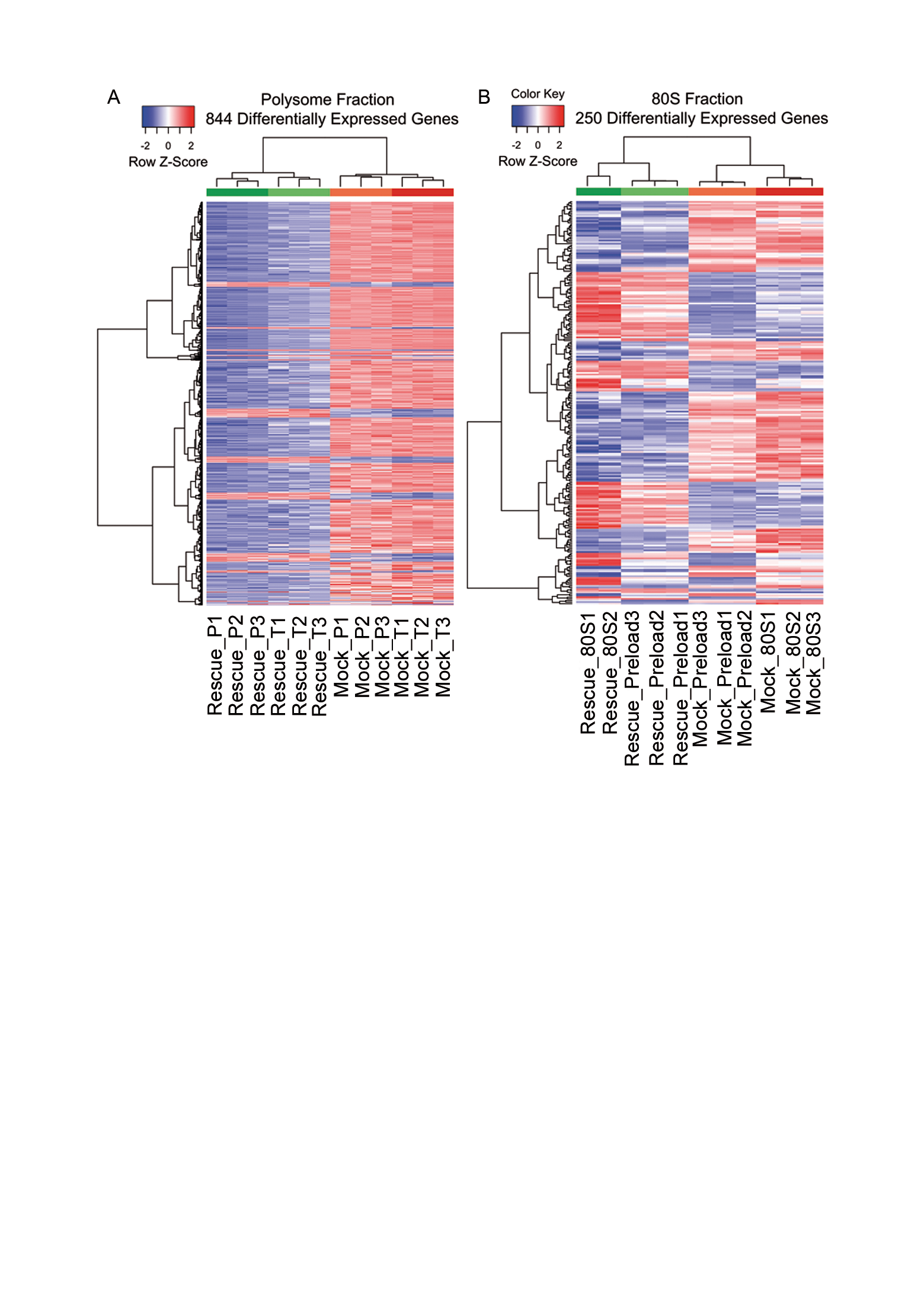

Supplement: S3 Fig — (A-B) Heat maps representing gene expression profile in polysomal and total fractions (biological replicates) for the pool of 844 genes identified as significantly changed in polysomes (A) and in 80S (B) and pre-load samples for the pool of 250 genes selected as significantly changed in 80S fraction. Values are represented as z-scores after rlog transformation. (TIF) [file pgen.1006552.s003.tif]

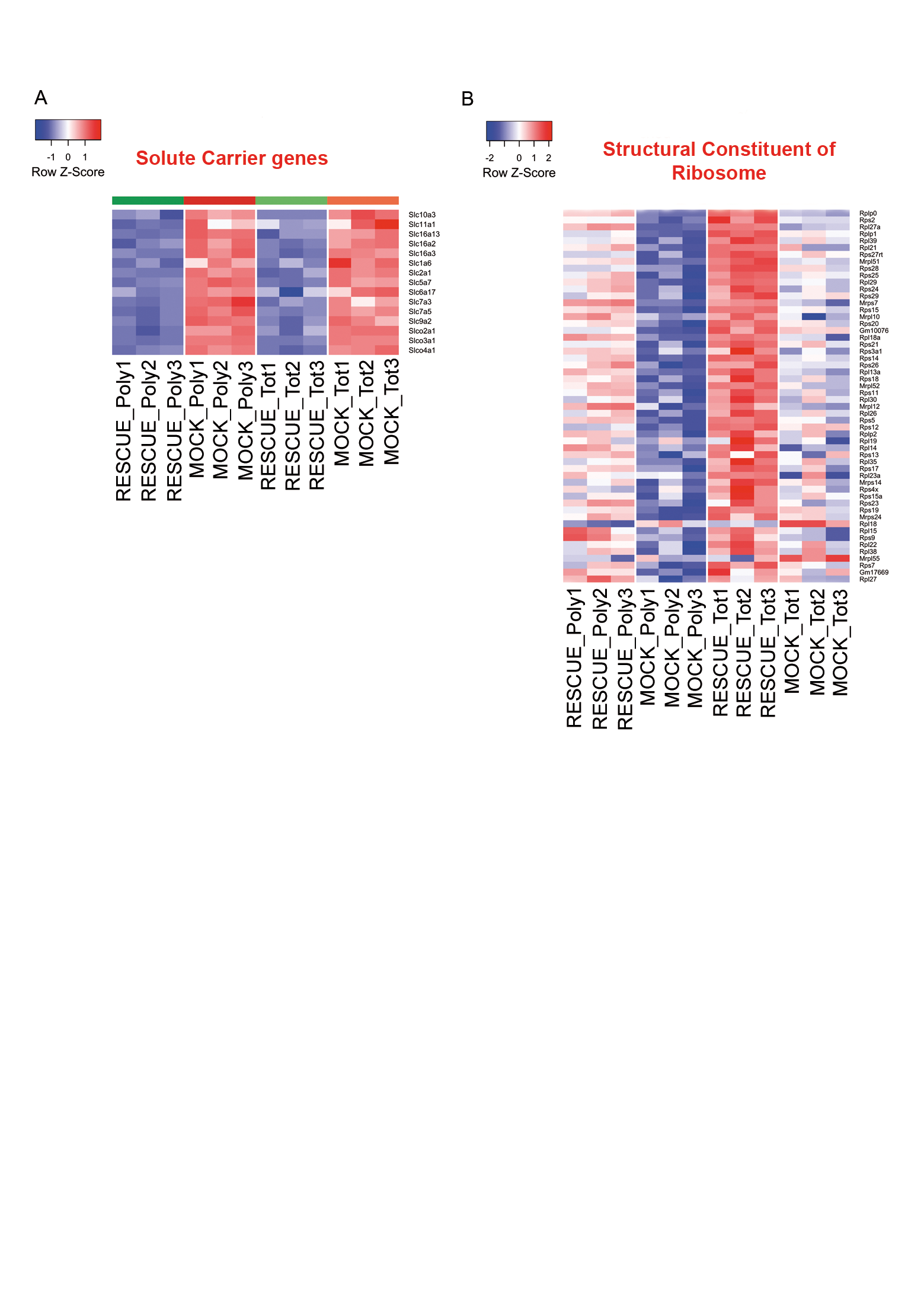

Supplement: S4 Fig — (A-B) Heat maps representing gene expression profile of mRNAs from the solute carrier genes class (A) and for the structural constituent of the ribosome (B). Values are represented as z-scores after rlog transformation. (TIF) [file pgen.1006552.s004.tif]

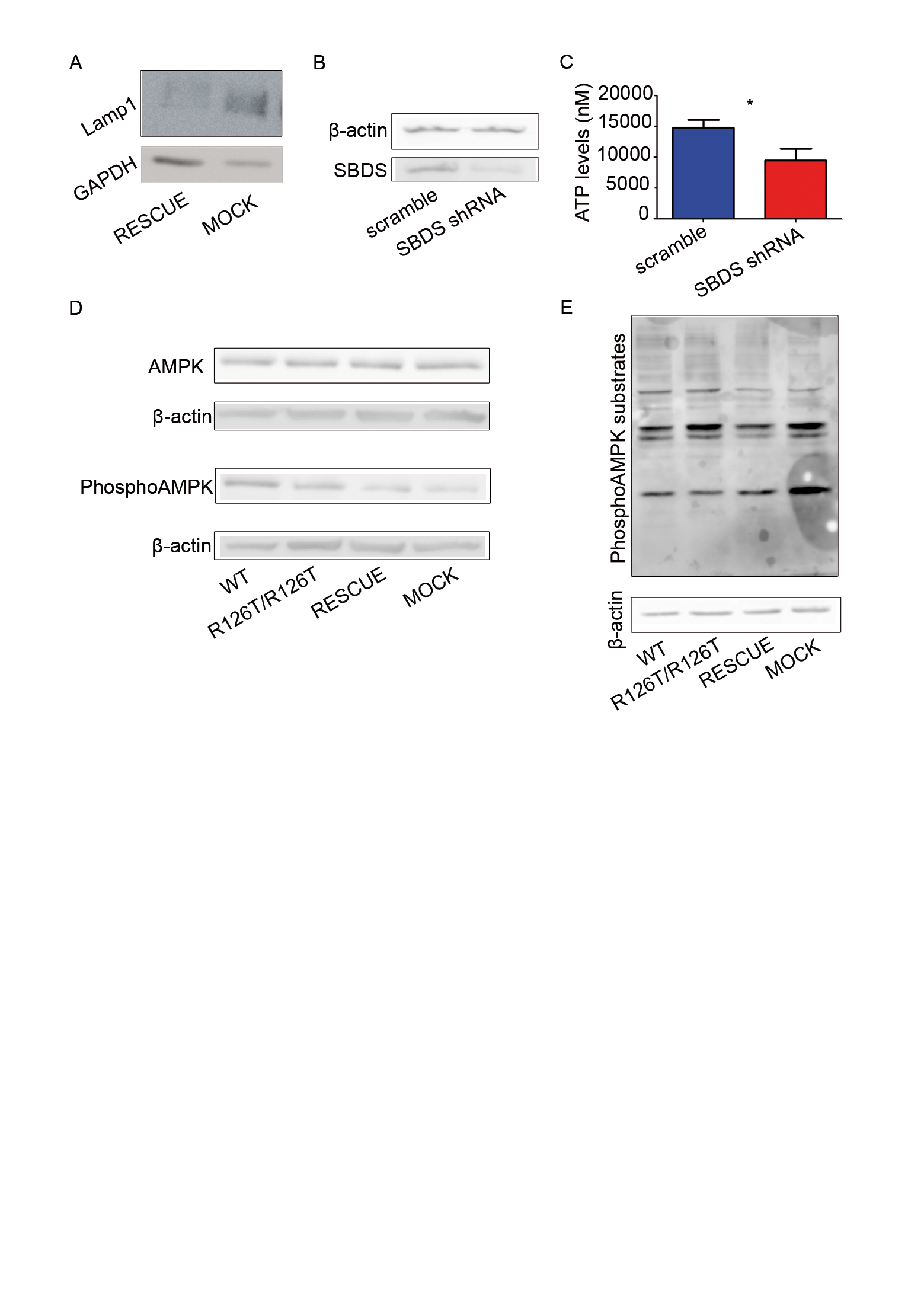

Supplement: S5 Fig — (A) Representative Western Blot showing increased levels of Lamp1 in SbdsMOCK cells respect to SbdsRESCUE MEFs. (B) Representative Western Blot showing the levels of SBDS protein in HEK-293T cells infected with the pFCY SBDS shRNA lentiviral vector (SBDS shRNA) or pFCY scramble vector (scramble). (C) ATP levels in SBDS shRNA HEK-293T cells were reduced when compared to control cells. Graphs represent the mean of values, error bars indicates standard deviation. Two-tailed t-test, paired (*P value≤0.05, ***P≤0.001). (D) Representative Western Blot showing that wt, SbdsR126T/R126T, SbdsRESCUE and SbdsMOCK MEFs have the same levels of AMPK and phosphoAMPK proteins. (E) Representative Western Blot showing that both SbdsR126T/R126T and SbdsMOCK MEFs have a mild increase in phosphoAMPK substrates compared to wild type and SbdsRESCUE cells. (TIF) [file pgen.1006552.s005.tif]

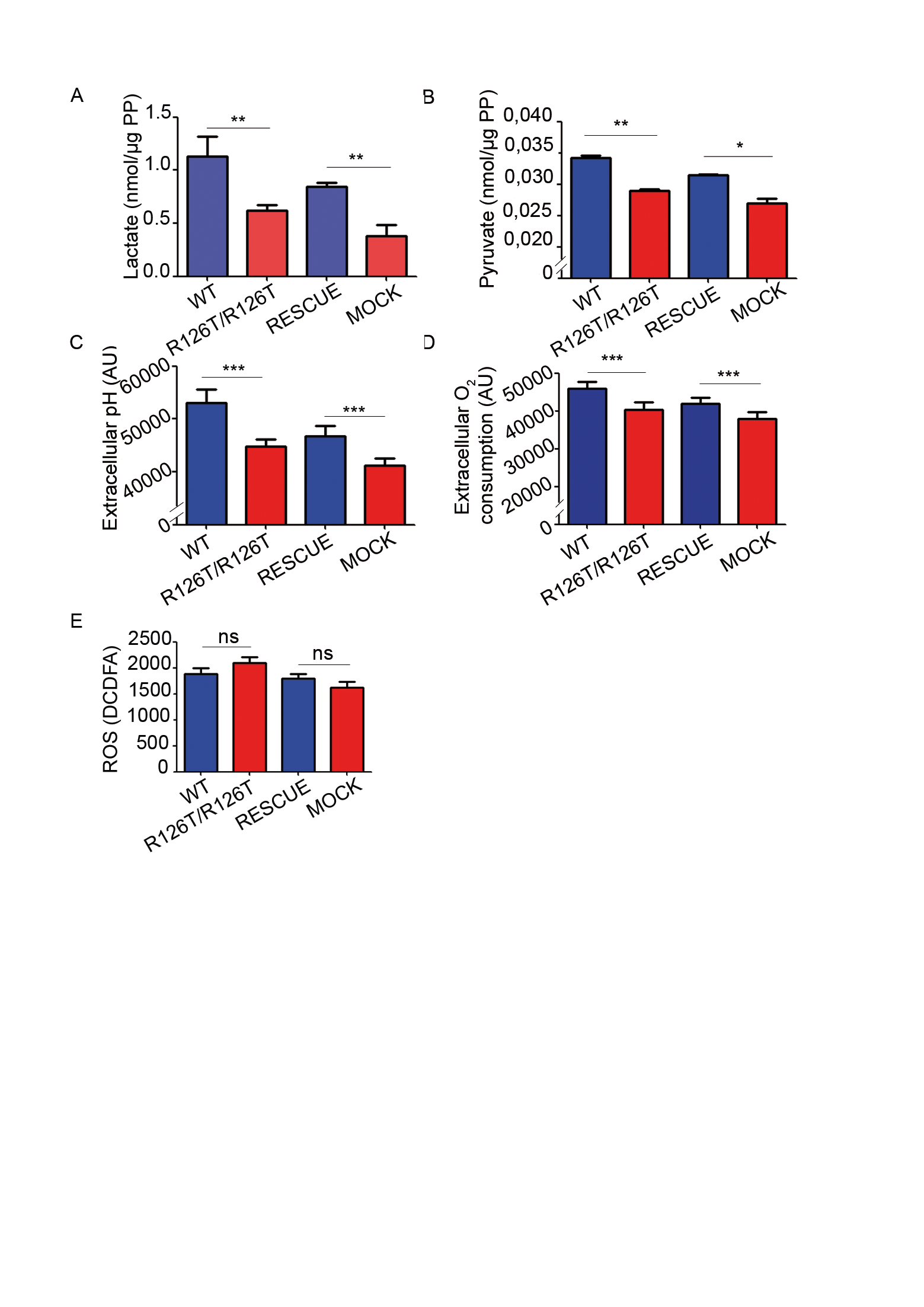

Supplement: S6 Fig — SbdsR126T/R126T and SbdsMOCK MEFs display a significant decrease in lactate (A) and pyruvate (B) levels, also confirmed by a reduction in glycolytic activity (C), measured with extracellular pH levels, respect to their controls wild type and SbdsRESCUE MEFs. SbdsR126T/R126T and SbdsMOCK MEFs display also a reduction in respiration (D), but ROS levels (E) remain unchanged. Graphs represent the mean of values, error bars indicates standard deviation. Two-tailed t-test, paired (*P value≤0.05, ***P≤0.001). (TIF) [file pgen.1006552.s006.tif]

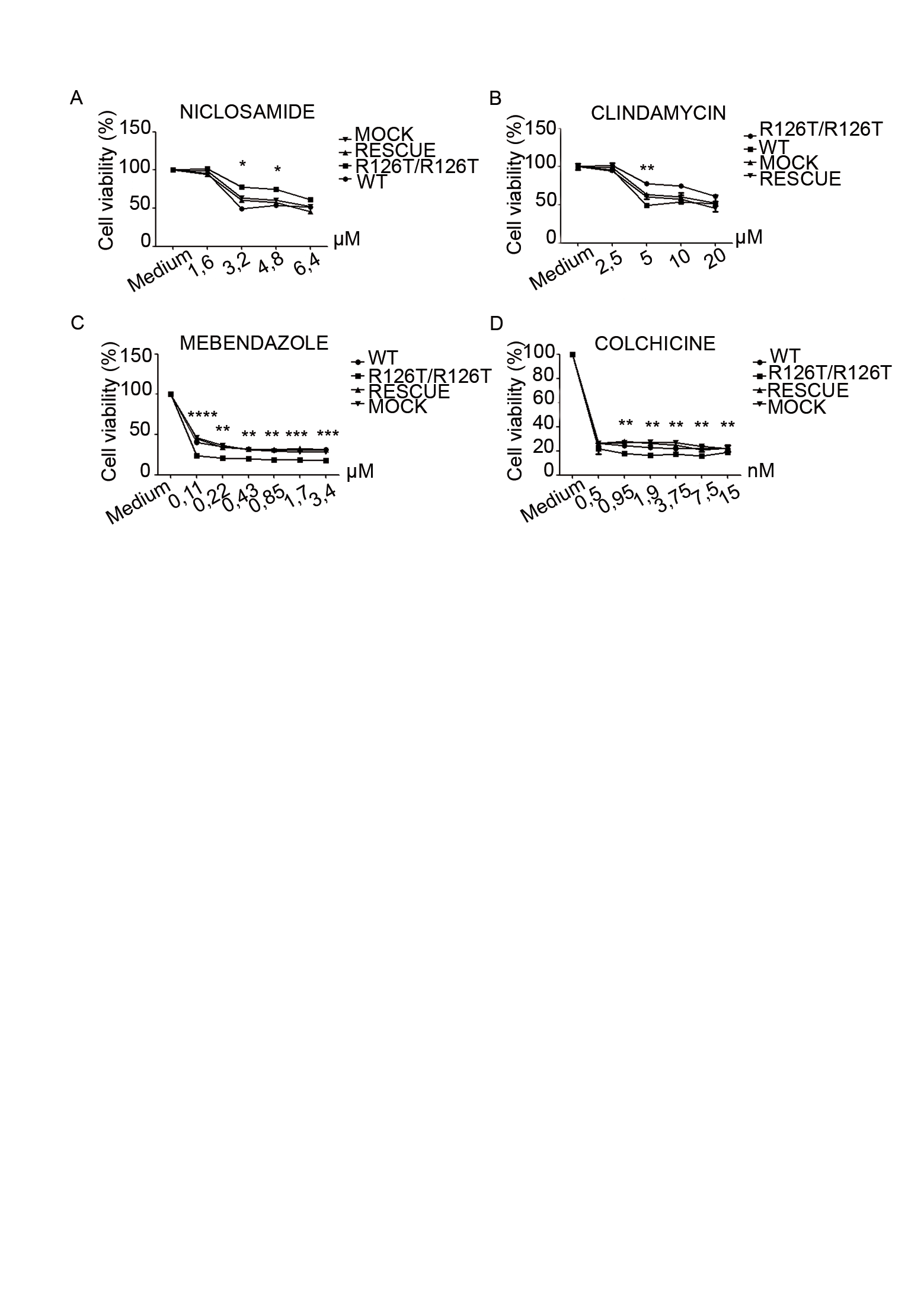

Supplement: S7 Fig — After the first large screening, molecules showing different activity in SbdsR126T/R126T have been selected for a second dose-response assay including the SbdsRESCUE. Four drugs confirmed the data on the parental cell lines but were not rescued, suggesting clonal variations or indirect effects. (A) Niclosamide. (B) Clindamycin. (C) Mebendazole. (D) Colchicine. (TIF) [file pgen.1006552.s007.tif]
